# Sensing the structural and conformational properties of single-stranded nucleic acids using electrometry and molecular simulations

**DOI:** 10.1038/s41598-024-70641-x

**Published:** 2024-09-04

**Authors:** Rowan Walker-Gibbons, Xin Zhu, Ali Behjatian, Timothy J. D. Bennett, Madhavi Krishnan

**Affiliations:** 1 Physical and Theoretical Chemistry Laboratory, Department of Chemistry, University of Oxford, South Parks Road, Oxford, OX1 3QZ UK; 2 The Kavli Institute for Nanoscience Discovery, Sherrington Road, Oxford, OX1 3QU UK

**Keywords:** Single stranded nucleic acids, Disordered molecules, Molecular electrostatics, Molecular dynamics, Physical chemistry, Biophysical chemistry

## Abstract

Inferring the 3D structure and conformation of disordered biomolecules, e.g., single stranded nucleic acids (ssNAs), remains challenging due to their conformational heterogeneity in solution. Here, we use escape-time electrometry (ET*e*) to measure with sub elementary-charge precision the effective electrical charge in solution of short to medium chain length ssNAs in the range of 5–60 bases*.* We compare measurements of molecular effective charge with theoretically calculated values for simulated molecular conformations obtained from Molecular Dynamics simulations using a variety of forcefield descriptions. We demonstrate that the measured effective charge captures subtle differences in molecular structure in various nucleic acid homopolymers of identical length, and also that the experimental measurements can find agreement with computed values derived from coarse-grained molecular structure descriptions such as oxDNA, as well next generation ssNA force fields. We further show that comparing the measured effective charge with calculations for a rigid, charged rod—the simplest model of a nucleic acid—yields estimates of molecular structural dimensions such as linear charge spacings that capture molecular structural trends observed using high resolution structural analysis methods such as X-ray scattering. By sensitively probing the effective charge of a molecule, electrometry provides a powerful dimension supporting inferences of molecular structural and conformational properties, as well as the validation of biomolecular structural models. The overall approach holds promise for a high throughput, microscopy-based biomolecular analytical approach offering rapid screening and inference of molecular 3D conformation, and operating at the single molecule level in solution.

## Introduction

The structural and conformational properties of nucleic acids have been thoroughly examined in a range of contexts using a variety of experimental techniques and we now have a detailed picture of their atomic structure, surrounding ion atmospheres in solution, the dynamics of hydration water and the complexes they form with proteins^1–6^. Whilst double-stranded (ds) DNA has been extensively studied due to its obvious importance as the “genetic blueprint”, far less is known about the structural properties of single stranded nucleic acids (ssNAs) in comparison. This is in large part due to the fact that ssNAs are flexible polymers, with an inherently disordered structure, and exist as heterogeneous conformational ensembles that may not be well represented by single ensemble-averaged 3D structures. Unlike folded biomolecules, e.g., globular proteins, ribozymes and riboswitches, disordered biomolecules often do not lend themselves well to high-resolution structural techniques such as X-ray crystallography and cryo-electron microscopy^7,8^. Yet knowledge of the molecular level conformation and local atomistic arrangement of ssNAs is important to characterise their interactions, e.g., protein-RNA interactions are important for CRISPR-Cas9 technologies and in driving phase separation and modulating the properties of biomolecular condensates^9–12^. Furthermore, the conformational properties of biologically significant homopolymeric motifs such as U/A tracts in ssRNA serve important functions in protein recognition, the regulation of gene expression, and the assembly of virus like particles^13–18^. ssDNA also finds extensive use as a therapeutic agent for a host of diseases, a platform for the development of vaccines and as a polymer in nucleic acid origami nanotechnology^19–22^. The conformational richness displayed by ssNAs holds the key to their diverse roles and interactions. The challenges and opportunities that lie in the problem of structure-conformation inferences on disordered biomolecules heightens the importance of developing new and orthogonal measurement modalities that in conjunction with other techniques may contribute to multi-parametric structural and conformational analyses^15,23^.

Experimental investigations into the structure of ssNAs thus far have largely relied upon techniques such as small angle X-ray scattering (SAXS), nuclear magnetic resonance (NMR) and single molecule Förster resonance energy transfer (smFRET)^15,24–27^. SAXS is a label-free method that has proven very powerful in examining ssNA conformation, revealing both local and global sequence dependent chain conformations that relate to base stacking affinities and the local ion atmospheres around nucleic acids^15,23,24^. A range of experimental studies have uncovered clear salt- and sequence-dependent trends in conformational properties, e.g., the persistence length, ${l}_{\text{p}}$, and radius of gyration ${R}_{\text{g}}$, of ssDNA were observed to decrease with an increasing salt concentration, and stiffer polymer conformations were inferred for poly-dA structures compared to -dT which was attributed to stronger inter-base stacking between adenine bases compared to thymine^26,28–31^.

**Fig. 1:**
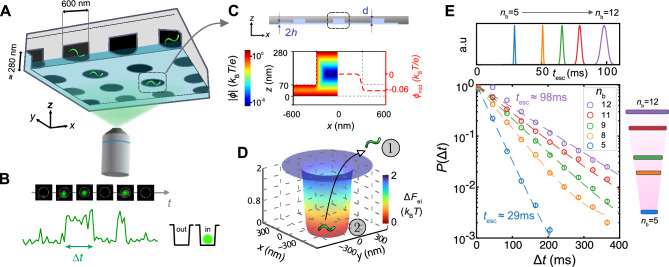
Measurement of the effective charge of ssNAs using ET*e*. (**A**) Schematic representation (not to scale) of ssNA molecules confined in an array of electrostatic fluidic traps and imaged using wide-field fluorescence microscopy. (**B**) A time course of optical images in a single trap displays the duration of a single recorded residence event of duration, $\Delta t$. (**C**) Graphical representation of the cross- section of a fluidic slit of height $2h \approx 70$ nm, with a pocket depth $d \approx 210$ nm (top). Below: Calculated electrostatic potential distribution, $\phi$, presented on logarithmic scale for a salt concentration ${c}_{0}=$ 1.2 mM and $h =$ 35 nm (left half-space). Line plot of midplane potential value, ${\phi }_{\text{mid}}$, in the vicinity of a single pocket (right half-space), displays a value $\Delta {\phi }_{\text{mid}}={\phi }_{\text{m}}\approx$ −0.06 ${k}_{\text{B}}T/e$ in the slit region, corresponding to an effective surface potential of ${\phi }_{\text{s}}\approx$ −2.2 ${k}_{\text{B}}T/e$. (**D**) A representative electrostatic energy landscape ($\Delta {F}_{\text{el}}$) for a single trap in this study showing the spatial states of the molecule in the slit (out: “1”) and pocket (in: “2”). (**E**) Probability density distributions $P\left(\Delta t\right)$ of escape times obtained from 3–5 min of imaging for short chain ssDNA fragments (bottom). All measurements were performed in devices with slits of depth $2h=$ 70 nm and surface potential value ${\phi }_{\text{s}}=$ $-1.91\,{k}_{\text{B}}T/e$, under in an electrolyte containing NaCl at a concentration $c_0=$ 0.6 mM ($\kappa h$ ≈ 5.5) and pH $=$ 7.5. Measured ${t}_{\text{esc}}$ values for oligomers of length ${n}_{\text{b}}=$ 5–12 reveal clear differences (top panel)$.$ The average molecular escape times, ${t}_{\text{esc}}$, measured effective charge values, ${q}_{\text{eff}}$, and number of escape events, $N$, are as follows: ${n}_{\text{b}}=5$: ${t}_{\text{esc}}=$ $29.16\pm 0.38$ ms (${q}_{\text{eff}}=-6.3\pm 0.082\, e$, $N=2.2\times {10}^{4}$), ${n}_{\text{b}}=8$: ${t}_{\text{esc}}=$ $50.82\pm 0.43$ ms (${q}_{\text{eff}}=-9.2\pm 0.077\,e, N=3.7\times {10}^{4}$), ${n}_{\text{b}}=9$: ${t}_{\text{esc}}=$ $65.68\pm 0.89$ ms (${q}_{\text{eff}}=-9.8\pm 0.13\, e, N=2.4\times {10}^{4}$), ${n}_{\text{b}}=11$: ${t}_{\text{esc}}=$ $79.24\pm 1.30$ ms (${q}_{\text{eff}}=-10.7\pm 0.175\,e, N=8.0 \times {10}^{4}$), ${n}_{\text{b}}=12$: ${t}_{\text{esc}}=$ $98.08\pm 2.3$ ms (${q}_{\text{eff}}=-11.9\pm 0.28\,e, N=3.0\times {10}^{4}$). Widths of presented Gaussian distributions of ${t}_{\text{esc}}$ arise predominantly from the statistical error on the fitted ${t}_{\text{esc}}$ values which are given approximately by ${t}_{\text{esc}}/\sqrt{N}$ (see “Methods”).

We recently pioneered escape time electrometry (ET*e*)—a field free, optical microscopy-based technique that measures electrostatic interaction energies between a charged molecule and a like-charged surface with high precision. Because the magnitude of electrostatic interactions carries a strong signature of molecular shape or 3D conformation, such measurements can offer a fundamentally different approach to inferring molecular structural information in solution^32–34^. The ET*e* method relies on the electrostatic fluidic trap to spatially confine charged molecules diffusing in solution in a geometrically tailored slit created by two like-charged parallel surfaces (Fig. 1A,C). Measurement of the time spent by individual molecules in the trapped state furnishes a high precision inference of the interaction energy between the molecule and the flanking slit surfaces, which in turn can be parametrized in terms of an effective charge, ${q}_{\text{eff}}$, of the molecular species, as previously described^32–38^. The effective charge of a molecule in solution is often smaller than the molecule’s structural charge, ${q}_{\text{str}}$, (given by the sum of charges carried by ionized structural groups and any structurally bound ions from the electrolyte), and its departure from the latter is captured by a renormalization factor given by $\eta =\frac{{q}_{\text{eff}}}{{q}_{\text{str}}}$. The greater the extent of charge renormalization in the molecule the smaller the value of $\eta$. For a highly ionized (acidic) object such as a nucleic acid molecule, $\eta$ is solely a function of the geometry of the molecular charge distribution or the 3D conformation of the molecule^33,35,39,40^.

Our definition of molecular effective charge is founded in electrostatic interaction free energies as previously described, and is in fact in good agreement with a range of other estimates of ${q}_{\text{eff}}$ based on different but related considerations^35,40–42^. For example ${q}_{\text{eff}}$ as defined in our work agrees reasonably well with analytical estimates for charged spheres and cylinders given in Ref.^40^ which we invoke here in order to provide physical intuition and understanding of the background of our approach. For charged cylinders of radius $R$ and length $l\gg {\kappa }^{-1}$ carrying a uniformly distributed charge of ${q}_{\text{str}}$, which describes polyelectrolytes such as DNA, Ref.^40^ suggests

1 $$\left|\frac{{q}_{\text{eff}}}{e}\right|=\left(\frac{l}{{l}_{\text{B}}}\right)\left(1-\frac{\text{ln}[\left|\frac{{q}_{\text{str}}}{e}\right|(\frac{{l}_{\text{B}}}{l})]}{2\text{ln}(\kappa R)}\right)$$

in the regime $\kappa R<1$ which corresponds to a typical polyelectrolyte immersed in an electrolyte of ionic strength ${c}_{0}\approx$ 1 mM. Here $e$ denotes the elementary charge, and ${\kappa }^{-1}\approx 0.304/\sqrt{{c}_{0}}$ nm is the Debye length which characterizes the spatial rate of decay of the electrostatic potential from a charged interface in an electrolyte containing only monovalent ions. We also have ${l}_{\text{B}}\approx 0.704$ nm, the Bjerrum length in water, which denotes the spatial separation at which two identical charges experience ${k}_{\text{B}}T$ worth of interaction energy, where ${k}_{\text{B}}$ is Boltzmann’s constant and $T$ is the absolute temperature. Equation (1) shows that (1) the renormalized linear charge density tends to the Manning limit of $1/{l}_{\text{B}}$ as $\kappa R\to 0$ which e.g., describes a line charge, and (2) since $l={n}_{\text{b}}b$, where ${n}_{\text{b}}$ is the number of charged monomers and $b$ the spacing between them, a precise measurement of ${q}_{\text{eff}}$ can be sensitive to the charge spacing of a uniform polyelectrolyte. We recently demonstrated measurements of the rise per basepair and radius of two forms of the DNA double helix using this principle, and will demonstrate the ability to make structural inferences on ssNAs in this study^33,34,38^. In general, as indicated in Eq. (1), the more compact the 3D charge distribution—or the higher the linear charge density of the molecular backbone in the case of nucleic acids—the greater the influence of charge renormalization, the lower the magnitude of ${q}_{\text{eff}}$ and hence the lower the value of $\eta$^35^.

It is also worth connecting the interpretation of our definition of ${q}_{\text{eff}}$ with readouts of experimental techniques like atomic emission spectroscopy (AES) and anomalous SAXS (ASAXS) which infer an excess concentration of counterions of type $i$, ${\Gamma }_{i}$, in the ion atmosphere, surrounding a charged molecule relative to the bulk solution^43^. Both ${q}_{\text{eff}}$ and ${\Gamma }_{i}$ provide descriptions of charge renormalization and derive from the same underlying physical quantity: the electrical potential distribution around a molecule in solution. In order to make a direct link between these two closely related quantities we have conducted an analysis for a negatively charged rod under the conditions of our present experiments and suggest the approximate relationship ${\Gamma }_{i}\propto {a}_{1}+{a}_{2}\eta$, where ${a}_{2}<0$ (see Supplementary Information Sect. S7). In general the parameters ${a}_{1}$ and ${a}_{2}$ in this equation depend on molecular structural charge, geometry and salt concentration. Importantly the given relationship captures the qualitative expectation that a lower magnitude of ${q}_{\text{eff}}$ (smaller $\eta$) entails a larger number of excess counterions in the molecule’s ion atmosphere, and will be often invoked in comparative discussions of results in this study with those from ion counting methods like AES.

Previous studies using electrometry have shown that measured values of ${q}_{\text{eff}}$ agree remarkably well with effective charge values calculated using Poisson-Boltzmann (PB) theory across a diverse range of molecules displaying distinct molecular conformations^32–35^. PB theory is widely known to be successful in describing the electrostatics of DNA, for instance in capturing the fraction of bound counterions and the nature of the surrounding ion distribution, predicting salt effects on the binding affinities of ligands and proteins to nucleic acids, and in modelling DNA adsorption behaviour to biological membranes and DNA-DNA interactions in supercoils^44–52^. A recent ET*e* investigation of DNA nanostructures of identical molecular weight but different 3D conformation illustrated the intimate relationship between 3D molecular conformation and the far-field electrostatic signature of a molecule, permitting various molecular 3D conformations to be readily differentiated according to their measured ${q}_{\text{eff}}$ values^33^. A related study focussing on the nucleic acid double helix demonstrated that high-precision ET*e* could further provide a route to determining higher resolution structural information such as the radius and the average rise per basepair of A- and B-form double helices characteristic of dsDNA and dsRNA. These measurements also furnished an experimentally inferred view of the interfacial hydration layer and the arrangement of ions at the molecular interface with the surrounding electrolyte^34^.

Following in the vein of high-resolution structural modelling techniques that compare experimental readouts with expectations based on computational modelling, in this study we undertake a comparison of measured ${q}_{\text{eff}}$ values with the theoretically expected effective charge, ${q}_{\text{calc}}$, for various ssNA species. In particular we employ a range of all-atom force fields including the standard Amber and CHARMM models and some next generation specialised ssNA forcefields such as DES-Amber, as well as a coarse-grained model—oxDNA. In order to relate our measurements of ${q}_{\text{eff}}$ to ssNA structural properties, we select a subset of representative model structures from each simulated conformational ensemble for nucleic acid molecules, and calculate their theoretically expected effective charge, ${q}_{\text{calc}}$, using our previously described PB free energy framework^35,38^. We then compare our measurements with modelling results and discuss the performance of each model and forcefield in capturing both our measurements as well as trends reported using other experimental techniques such as SAXS and AES.

The first part of the study focuses on the experimental approach, demonstrating the ability to measure molecular effective charge on small ssNA fragments. We start by probing an experimental regime where there is not much influence of molecular 3D conformation on ${q}_{\text{eff}}$ and show that this is indeed reflected in computationally modelled values based on MD conformations of short fragments. Next we perform ET*e* on longer ssNA fragments (30 and 60 base length) and show that differences in effective charge between various homopolymeric stretches of NAs clearly emerge in measurement. In comparing measured effective charge values with those computed from molecular models, in general we found that ${q}_{\text{eff}}$ carried contributions both from global conformation features and local structural information such as charge density along the molecular backbone. Whilst this is expected on qualitative grounds, a systematic quantitative delineation of the relative contributions is likely to be highly challenging. We also found that absolute comparisons between ${q}_{\text{eff}}$ and ${q}_{\text{calc}}$ are limited by our current level of experimental inaccuracy, which we estimate at about 10% as described later. We therefore do not attempt at this stage to perform ensemble optimization and refinement on the modelled molecular conformations in order to achieve agreement with experiments, as is more common practice in studies involving established structural techniques such as SAXS^15,23^. Nonetheless, relative trends in ${q}_{\text{eff}}$ values for different species measured under the same conditions are robust to measurement inaccuracies and could be used for comparisons with corresponding trends in computational models. In general our results will show that we did not obtain perfect agreement with any particular molecular model, although we did note that specific models such as DES-Amber and oxDNA performed well in capturing certain measurement trends. Finally, we also demonstrate that line charge models may provide a simple route to parametrising measured differences between molecular species.

We begin by briefly describing the ET*e* method to measure electrostatic interaction energies and infer corresponding effective charge values for ssNAs in solution. We measure the average residence time or escape time, ${t}_{\text{esc}}$, of a molecular species in a landscape of electrostatic traps created by the geometric modulation of the gap between two charged plates in solution (Fig. 1). The escape time depends exponentially on the effective charge of the molecule, ${q}_{\text{eff}}$, which can be then be inferred from measured ${t}_{\text{esc}}$ values as described previously^32–34,36,37,53^. We work in the electrostatic regime corresponding to $\kappa h\approx$ 5.5–8. Here $2h=70-75$ nm is the separation between the charged surfaces in the slit region of an ET*e* measurement device (Fig. S2). The salt concentration ${c}_{0}$ lies in the range $0.6-1.2$ mM in this work.

We consider a series of oligomers of length between 5 and 12 bases, and two longer fragments of length 30 and 60 bases. We explore how the molecular effective charge of ssNAs in solution varies as a function of both number of bases, ${n}_{\text{b}}$, as well as sequence. In particular, for ${n}_{\text{b}}$ = 30 and 60, we examine ssNA species carrying the same amount of total structural charge but differing in nucleotide sequence, namely poly-dT, -dA, -rU and a mixed sequence case. In tandem we use a PB interaction free energy framework described previously to calculate effective charge values, ${q}_{\text{calc}}$, for model structures of ssNAs generated using Molecular Dynamics (MD) simulations. We then compare measurements of ${q}_{\text{eff}}$ with the computed results. The conformational properties of the homopolymeric motifs in particular have been extensively studied using different experimental techniques and therefore serve as well characterised test cases with which to compare our results. Comparing our measurements with computationally generated ${q}_{\text{calc}}$ values, we demonstrate how ET*e* measurements of the effective molecular charge in solution can serve to infer aspects of molecular structure and conformation, and may also provide an additional physical dimension using which to validate structural models of charged biomolecules in solution.

## Results and discussion

### Molecular effective charge values for short chain length ssNA fragments

We first measured the escape dynamics of a series of short chain length ssDNA fragments (${n}_{\text{b}}=$ 5–12) diffusing in a lattice of electrostatic fluidic traps (see Fig. 1). The oligomeric species displayed distinct average escape times, ${t}_{\text{esc}}$, with the smallest ${n}_{\text{b}}$ = 5 fragment exhibiting an escape time of ${t}_{\text{esc}}\approx$ 29 ms and the longest fragment with ${n}_{\text{b}}$ = 12 a value of ${t}_{\text{esc}}\approx$ 98 ms (Fig. 1E). The measured values of ${t}_{\text{esc}}$ were then converted to values of molecular effective charge, ${q}_{\text{eff}}$ (see “Methods” and Refs.^32–34,36,37,53^). A calibration measurement using 60 basepair dsDNA—a species that has been previously thoroughly characterized in electrometry—was used to determine the value of the surface potential of the slit, ${\phi }_{\text{s}}$, which is a priori unknown (see “Methods”)^32,34^.

**Fig. 2:**
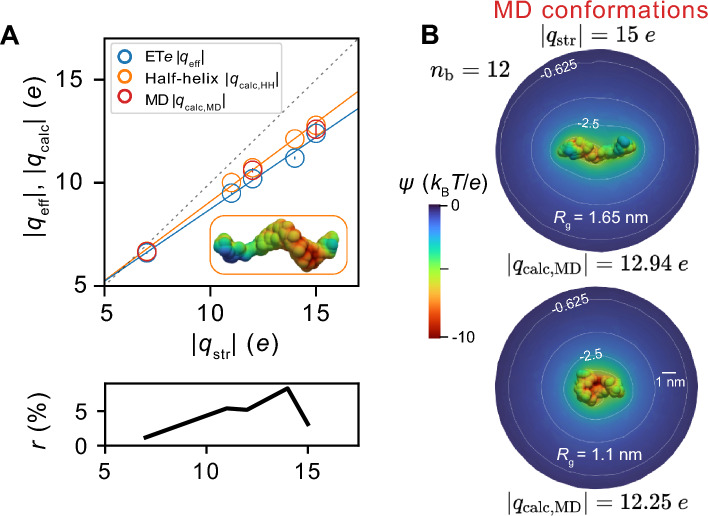
Molecular modelling of short chain ssNAs (${n}_{\text{b}}=$ 5–12) and comparison of experimental measurements of molecular effective charge ($|{q}_{\text{eff}}|$) with calculations ($|{q}_{\text{calc}}|$). (**A**) Plot of the molecular effective charge |${q}_{\text{eff}}|$ as measured by ET*e* (blue symbols), calculated for model half-helix structures (|${q}_{\text{calc},\text{HH}}|$, orange symbols) and for model conformations taken from MD simulations (${|q}_{\text{calc},\text{MD}}|$, red symbols) vs. molecular structural charge ${|q}_{\text{str}}|$. The simulated structures are free of dye molecules but the effective charge of two ATTO dye molecules is added post-calculation to enable comparison with the experimental measurements (see “Methods” and Figs. S1, S3). Error bars on ${|q}_{\text{eff}}|$ arise predominantly from the statistical error on the fitted ${t}_{\text{esc}}$ values. The error bars for the MD data points represent extrema values of the effective charge of structures across the ${R}_{\text{g}}$ range of the molecule observed in simulations. Dashed grey line represents $\eta =1$, presented for reference. Lower panel: Plot of the percentage difference, $r$, between calculations of effective charge for model half-helix structures, |${q}_{\text{calc},\text{HH}}|$, and experimental measurements ${|q}_{\text{eff}}|$. (**B**) Representative electrostatic potential distributions surrounding an ${n}_{\text{b}}=$ 12 oligomer (${|q}_{\text{str}}|=$ 15 *e*) obtained by solving the PB equation for 3D atomic charge distributions of conformations corresponding to extrema values of ${R}_{\text{g}}=$ 1.65 nm (top) and ${R}_{\text{g}}=$ 1.1 nm (bottom) taken from an MD simulation using the DES-Amber forcefield. Electrostatic potential contours are shown at $\phi =$ − 5, − 2.5, − 1.25 and − 0.625 ${k}_{\text{B}}T/e$. The difference in |${q}_{\text{eff}}|$ between the two structures is $\approx$ 0.7 *e* or $<$ 6% of the mean value of 12.6 *e*.

We then compared our measurements with effective charge values for simulated molecular structures, ${q}_{\text{calc}}$, calculated as described previously^33–35,38,54^. Note that each molecular species (apart from the ${n}_{\text{b}}$ = 5 case) is characterised by a value of ${q}_{\text{str}}=-\left({n}_{b}+3\right)e$, which results from accounting for the additional charges of the attached fluorescent dyes (see “Methods” and Figs. S1, S3). We found close agreement (within 5*%* on average) between the effective charge values computed for model ‘half-helix’ structures, ${q}_{\text{calc},\text{HH}},$ (orange data points) and the experimentally measured data (blue data points) (see Fig. 2A). Performing 0.5 $\upmu$s long MD simulations (employing the DES-Amber forcefield) on these short chain molecules permitted their configurations to depart from their initial helical structure, yielding a conformational ensemble of structures characterised by a spectrum of ${R}_{\text{g}}$ values and therefore a range of calculated ${q}_{\text{calc},\text{MD}}$ values. Calculations of the effective charge for conformations corresponding approximately to the minimum and maximum ${R}_{\text{g}}$ values obtained from MD simulations (see “Methods” and Fig. S2 for details) revealed a small amount of variation in their ${q}_{\text{calc},\text{MD}}$ values, reflecting the conformational heterogeneity (associated error bars of the MD structure data points in Fig. 2A). In order to enable a quantitative comparison of calculated values with experimental readouts, the effective charge contributed by the covalently attached fluorescent dye molecules was added *a posteriori* to the effective charge calculated for dye-free molecular conformations (see “Methods” and Fig. S3). Importantly, for the series of short nucleic acid molecules, we found that the measurements revealed ${|q}_{\text{eff}}|\approx {|q}_{\text{str}}|$. In the regime where a typical length scale describing the extent of the molecule, such as the polymer contour length ${l}_{\text{c}}$, is smaller than the Debye length, i.e., ${l}_{\text{c}}\approx 5$ nm $<{\kappa }^{-1}\approx 10$ nm, the ‘far-field’ electrostatic potential distribution resembles that of a sphere and charge renormalization vanishes, as recognised in previous theoretical work^39,40^. Under our present experimental conditions ($\kappa {l}_{\text{c}}<$ 1), the conformational properties of very short fragments of ssDNA are not expected to significantly impact the measured charge renormalization values, therefore implying $\eta \approx$ 1. Indeed, for 5-base ssDNA, for example, we measure $\eta$ = 0.94*.* Thus for short nucleic acids our present measurements are rather insensitive to the precise 3D arrangement of the charge in the molecule, since under the conditions of the measurement, the spatial extent of the molecular charge distribution under consideration is smaller than the Debye length. Figure 2B displays the contours of electrostatic potential around molecular structures corresponding approximately to the minimum and maximum ${R}_{\text{g}}$ for an ${n}_{\text{b}}=12$ polymer from MD, which can be seen to resemble one another a short distance away ($\sim 0.5{\kappa }^{-1}$) from the molecule, with the potential distribution appearing similar to that of a small sphere for smaller values of ${n}_{\text{b}}$. The two conformations are characterised by a difference in ${q}_{\text{calc}}$ of 0.7 *e* or less than 6% of the mean value of 12.6 *e*. For this reason, ${q}_{\text{calc}}$ values calculated for model structures consisting of only a half-helix, without any higher-order structural information, find good agreement both with MD-simulated conformations as well as with the experimental data. Thus, although little molecular structural or conformational insight can be gleaned in this electrostatic regime (${l}_{\text{c}}< {\kappa }^{-1})$, the results demonstrate sensitivity of the measurement to the addition of just a single nucleobase. Note that the measurement of 100–1000 molecules per species in this study yields high-precision measurements of ${q}_{\text{eff}}$ (measurement imprecision $\approx$ 0.1–1%), which should enable small structural differences between species to be discerned, as described later. Although highly sensitive to relative differences between species, we expect that absolute values of the measurements reported here may reflect an inaccuracy of up to 10%, which arises from a number of different experimental sources such as sample purity, and the influence thereof on the fitting procedure to extract ${t}_{\text{esc}}$ values, as well as accuracy limits on the value of ${q}_{\text{eff}}$ of the calibration molecule (see “Methods”). This can however be improved upon in future such that the accuracy of the method better reflects the precision. The inaccuracy of $\approx$ 10% on ${q}_{\text{eff}}$ in this work nonetheless does not preclude comparisons with computational estimates of the same quantity under conditions where ${q}_{\text{eff}}$ and ${q}_{\text{calc}}$ are more sensitive to the 3D charge distribution in the molecule, i.e., ${l}_{\text{c}}> {\kappa }^{-1}$, as described in the following section.

### Sequence dependence of effective charge values for longer chain ssNAs

Inspired by a range of X-ray scattering studies, smFRET, and force extension measurements that have reported structural differences between homopolymeric sequence ssNAs composed of an identical number of bases, we turned our attention to examining differences in homopolymeric ssNAs. The precision offered by ET*e* and the sensitivity of the underlying physics to the 3D charge distribution in the molecule may indeed offer the prospect of using effective charge to detect sequence dependent differences in molecular structure and/or conformation. Note that when measurements on various molecular species are performed under identical conditions, as defined by the same measurement device and solution conditions, the measurement precision ($\le$ 1% in this study) effectively becomes the relevant quantity in determining the ability of the approach to discriminate between different molecular states, conformations or species.

**Fig. 3:**
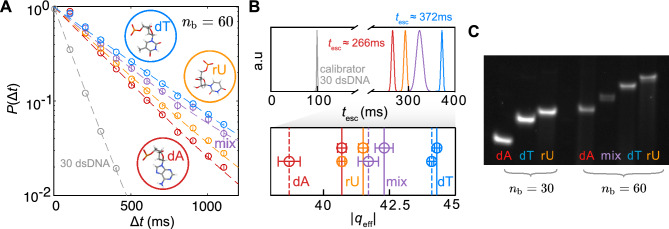
ET*e* detects composition differences between ssNA oligos. (**A**) Probability density distributions $P\left(\Delta t\right)$ of residence times for measurements on single stranded homopolymers poly-dT (blue), -dA (red), a mixed DNA sequence (purple) and RNA poly-rU (orange), shown here for the fragment length ${n}_{\text{b}}=60$, and for the calibrator molecule, 30dsDNA (grey) (see “Methods”). Atomic representations of the nucleotide bases are shown as insets. (**B**) Measured ${t}_{\text{esc}}$ values for the data presented in (**A**) (upper panel). Two datasets of ${|q}_{\text{eff}}|$ values inferred from the measured data (solid and dashed vertical lines), for the ${n}_{\text{b}}=60$ ssNA polymers (lower panel). Widths of presented Gaussian distributions of ${t}_{\text{esc}}$ and error bars on ${|q}_{\text{eff}}|$ arise predominantly from the statistical error on the fitted ${t}_{\text{esc}}$ values in (**A**). All measurements were performed in devices with slits of depth $2h$ = 75 nm and surface potential value ${\phi }_{\text{s}}$ = −$2.24\,{k}_{\text{B}}T/e$, under similar electrolyte conditions NaCl $=$ 1.2 mM, pH $=$ 7.5. The average molecular escape times, ${t}_{\text{esc}}$, measured effective charge values, ${q}_{\text{eff}}$, and number of escape events $N$ for the given measurement dataset are as follows: poly-dT: ${t}_{\text{esc}}=$ $372.01\pm 2.13$ ms (${q}_{\text{eff}}=-44.3\pm 0.0923\,e, N=1.1\times {10}^{5}$), poly-dA: ${t}_{\text{esc}}=$ $265.79\pm 2.62$ ms (${q}_{\text{eff}}=-40.7\pm 0.165\, e, N=6\times {10}^{4}$), mixed sequence: ${t}_{\text{esc}}=$ $323.73\pm 6.864$ ms (${q}_{\text{eff}}=-42.3\pm 0.342\,e, N=5.2\times {10}^{3}$), poly-rU: ${t}_{\text{esc}}=$ $293.11\pm 2.94$ ms (${q}_{\text{eff}}=-41.5\pm 0.165\,e, N=5.8\times {10}^{4}$). (**C**) Gel electrophoresis of ssNA samples for ${n}_{\text{b}}$ = 30 and 60 (see Fig. S6 for details). The electrophoretic mobility follows poly-dA > mixed sequence >  -dT >  -rU$.$

We performed experiments on ssNA species of two different lengths (${n}_{\text{b}}$= 30 and ${n}_{\text{b}}$ = 60) and explored the effect of nucleic acid sequence alone on the measured effective molecular charge. The measurement regime corresponds to ${l}_{\text{c}}>{\kappa }^{-1}$ where $\kappa {l}_{\text{c}}\approx$ 1.7 for 30 base and $\kappa {l}_{\text{c}}\approx$ 3.4 for 60 base ssDNA, assuming an approximate contour length per base value ${b}_{\text{c}}$ = 0.5 nm. In this regime we expect substantial charge renormalization ($\eta$<1), in contrast to the measurements performed on short ssNA fragments. Indeed we found significant charge renormalization corresponding to $\eta \approx$ 0.6–0.8 in general (e.g., $\eta \approx$ 0.8 and $\eta \approx$ 0.7 for 30 and 60 base poly-dT respectively), and differences in measured ${q}_{\text{eff}}$ of approximately 5% between 30 base poly-dT and poly-dA, and 10% between 60 base poly-dT and poly-dA. For instance, the measured difference in ${q}_{\text{eff}}$ between poly-dT and poly-dA homopolymers is $\approx$ 2* e* and $\approx$ 4.5 *e* for the ${n}_{\text{b}}$= 30 and ${n}_{\text{b}}$ = 60 length chains respectively. These values reflect inter-species disparities in effective charge that are much larger than measurement precisions of approximately 0.5% and 1% for ${n}_{\text{b}}$ = 30 and ${n}_{\text{b}}$ = 60 respectively. Importantly, the measured order of $\left|{q}_{\text{eff}}\right|$ follows poly-dT > mixed sequence > -rU $\ge$ -dA and implies different average molecular conformations of the homopolymers (Fig. 3A,B). We attribute the sensitivity of the readout to molecular structural detail to the fact that in the regime ${l}_{\text{c}}>{\kappa }^{-1}$, characterising 60 base fragments, higher order multipoles of the molecular charge distribution make a greater contribution to the electrical potential in the far-field, i.e., at large distances from the molecule, $h>3{\kappa }^{-1}$^55,56^. This accentuates the sensitivity of the electrical free energy measurement to molecular 3D conformation and highlights the ability of the ET*e* technique to sense spatial features of molecular charge density distributions.

We further noted good qualitative agreement in comparing trends in molecular effective charge and conformation deduced using electrometry with those inferred from other experimental techniques. The measured ${q}_{\text{eff}}$ values for poly-dA and poly-rU are significantly smaller than that of poly-dT indicating higher density charge distributions that are subject to greater charge renormalization (smaller $\eta$), compared to poly-dT (e.g. $\left|{q}_{\text{eff}}\right|$= 44.1 *e,* 38.7 *e* and 40.7 *e* for poly-dT, -dA and -rU, corresponding to $\eta$ values 0.70, 0.61, 0.65 respectively in a given measurement dataset)^24^. Techniques such as ASAXS and atomic emission spectroscopy (AES) directly quantify the number of excess ions in the ion atmosphere surrounding nucleic acids and therefore also report on a molecule’s effective charge in solution^43,57–59^. Plumridge et al. have conducted AES measurements of NA homopolymers and reported values for the excess number of Na^+^ counterions per phosphate to be $\approx$ 0.68, 0.71 and 0.83 for poly-dT30, -dA30 and -rU30 respectively, implying a smaller magnitude of ${q}_{\text{eff}}$ for poly-dA and -rU compared to poly-dT (see Eq. (1) and Supplementary Information Sect. S7)^15,60^. In accordance with these results, SAXS measurements found that poly-dT polymers were associated with the fewest excess ions whereas other oligonucleotides considered, which had T bases substituted with A, G or C, were all associated with a larger number of excess counterions^61^. Furthermore, an AES study comparing 50 base poly-rU and -dT ssNA homopolymers implied a smaller magnitude of ${q}_{\text{eff}}$ for RNA compared to DNA, and attributed this disparity to an increased polymer charge density brought about by the smaller inter-charge spacing for RNA^62^. We also note here that the counterion excess ${\Gamma }_{+}$ as reported by AES measurements can be less sensitive to the charge density of a rigid rod model of ssDNA than ${q}_{\text{eff}}$, particularly at low ionic strengths and in relation to the measurement precision of each method (see Supplementary Information Sect. S7 for further details). For example, the magnitude of the difference in ${\Gamma }_{+}$ between poly-dA and poly-dT of around 4% in AES measurements can be seen to correspond to $>$ 10% difference in $\eta$ (see Fig. S7)^60^. Furthermore, our measured differences in ${q}_{\text{eff}}$ between molecular species are much larger than the typical measurement imprecision and immediately suggest differences in the 3D distribution of charge in the various homopolymers that are qualitatively consistent with expectations from ion counting studies. In summary, ET*e* measurements of molecular effective charge in solution indicate that |${q}_{\text{eff}}|$ values follow the ordering poly-dT > mixed sequence > -rU $\ge$ -dA and are in agreement with AES studies. The ssNAs under consideration also display clear but different qualitative trends in their electrophoretic mobilities in polyacrylamide gels which may be more challenging to interpret and are discussed further in Supplementary Information Sect. S5 (Fig. 3C). Furthermore, inferences on molecular conformation from ET*e* in combination with molecular modelling analyses are in some cases supported both by experimental smFRET and SAXS measurements, as described in the following section^24^.

### Comparing electrometry measurements with the results of computational molecular modelling

#### General considerations

**Fig. 4:**
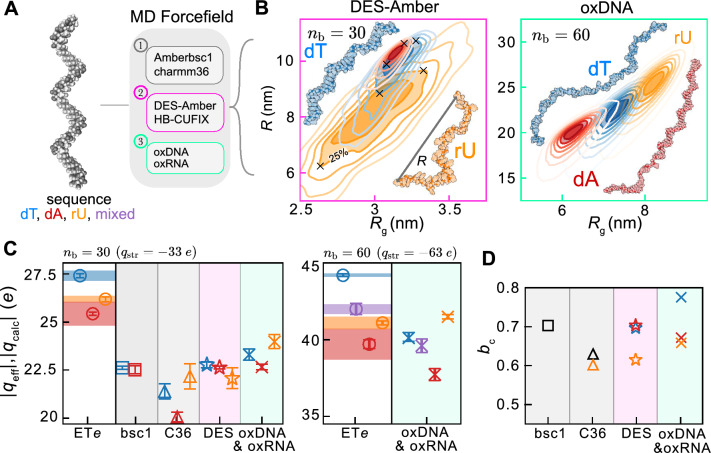
Molecular modelling and calculation of theoretical effective charge values for long chain ssNAs (fragment lengths ${n}_{\text{b}}$= 30 and 60) (**A**) Molecular model of the initial ‘half-helix’ configuration used for MD simulations employing conventional all-atom forcefields Amberbsc1 and CHARMM36, specialised ssNA DES-Amber and HBCUFIX forcefields and the coarse grained oxDNA and oxRNA models. (**B**) Simulated conformational landscapes of ssNA species (poly-dT—blue, poly-dA—red, poly-rU—orange), parametrised in terms of the polymer radius of gyration, ${R}_{\text{g}}$, and end-to-end distance, $R$. Each contour encloses a 12.5th percentile of structures. $R$ vs. ${R}_{\text{g}}$ landscapes are shown for the DES-Amber (left) and oxDNA models (right) for ${n}_{\text{b}}=$ 30 and ${n}_{\text{b}}=$ 60 respectively (inset: representative structures with the most likely $R$, ${R}_{\text{g}}$ combination). The $R$ vs. ${R}_{\text{g}}$ landscapes for ssNAs modelled with the other forcefield descriptions are shown in Fig. S5. (**C**) Comparison of the effective charge values, ${|q}_{\text{calc}}|$, for long chain ssNA structures (${n}_{\text{b}}=$ 30 and 60) obtained via molecular modelling approaches, with experimentally measured ${|q}_{\text{eff}}|$ data (open circles). Measurements (left column, circular symbols and associated measurement precision error bars) represent the average values of two measurement datasets. Horizontal shaded bands depict the range of $\left|{q}_{\text{eff}}\right|$ values across two measurement datasets. $\left|{q}_{\text{calc}}\right|$ values of representative model ssNA structures (right columns) carry upper and lower bounds that correspond to structures with the maximum and minimum $R$ values contained within the most likely 25th percentile of structures (corresponding to black crosses in (**B**)). (**D**) Contour length per base, ${b}_{\text{c}}$, values calculated from molecular simulations employing different forcefield descriptions, and where ${b}_{\text{c}}$ is calculated as the average inter-phosphate distance for all atom models or as the average inter-backbone site distance for oxDNA models. Black symbols denote values for both poly-dT and poly-dA sequences that share the same ${b}_{\text{c}}$ value.

Because the ET*e* readout is highly amenable to computational modelling, we embarked on an analysis that sought to quantitatively compare and relate the measurements with predictions from molecular modelling approaches. We therefore generated a host of representative ssNA structures from MD simulations employing various forcefield descriptions. The conformational free energy landscape of the molecule obtained from MD simulations was characterised by the radius of gyration ${R}_{\text{g}}$ and end-to-end distance parameter $R$ (Fig. 4B). We then determined ${q}_{\text{calc}}$ values for a pool of representative structures using our PB electrostatics framework (see “Methods”). We typically worked with a subset of structures for each case that included the most likely conformation as well as structures corresponding to the maximum and minimum $R$ values on the 25^th^ percentile contour of structures in ${(R}_{\text{g}}, R)$ parameter space (black crosses in Fig. 4B), furnishing a range of possible effective charge values for a given molecular species. Although the measured sequence-dependent differences in ${q}_{\text{eff}}$ are more significant for ${n}_{\text{b}}=$ 60 compared to ${n}_{\text{b}}=$ 30, we restricted all-atom simulation studies to 30-base oligomers owing to the significantly lower computational cost.

In keeping with qualitative expectations, we found in general that model structures with larger values of (${R}_{\text{g}},R$) gave rise to a larger magnitude of ${q}_{\text{calc}}$, and vice versa (error bars in Fig. 4C display the range of calculated values for each case). Despite the fact that we are only able to report all-atom simulation results for ${n}_{\text{b}}=$ 30 where the sensitivity of effective charge to molecular properties is poorer under the present experimental conditions, and measured disparities between the molecular species are therefore smaller, it must be noted that percentage changes in ${q}_{\text{calc}}$ are small compared to the corresponding percentage changes in ${R}_{\text{g}}$ and $R$ (e.g. $\approx$ 10% and 20% change in ${R}_{\text{g}}$ and $R$ respectively resulted in a 1.5% difference in ${q}_{\text{calc}}$ for poly-dT modelled with DES-Amber). Overall, we found that model structures for different molecular species, e.g., poly-dT, -dA and -rU, that are characterised by very similar values of ${(R}_{\text{g}},R)$, also had very a similar ${q}_{\text{calc}}$. However, we also found that this is not always the case, and there were instances where we noted more substantial differences ($\approx$ 2–3%) in ${q}_{\text{calc}}$ for model structures of different species characterised by the same ${(R}_{\text{g}},R)$. Therefore, the ET*e* readout appears to carry information reflecting both the global properties of the charge distribution, as captured for instance by ${(R}_{\text{g}},R)$, as well as local properties such as the charge density along the backbone of the molecule, and it is challenging to clearly quantify the relative magnitude of these contributions to the overall molecular effective charge.

#### Molecular dynamics simulations: the Amber and CHARMM forcefields

Of the available classical all-atom models for simulating nucleic acid dynamics, the parmbcs1 model of the Amber forcefields and the CHARMM36 forcefield represent the state of the art, and have undergone significant rounds of improvement since their inception^63^. However, these forcefields were originally parametrised for dsDNA and are therefore known to have shortcomings in modelling ssDNA^21,64^. Here we discuss theoretical molecular effective charge values obtained for ssNA structures taken from all-atom MD simulations performed with the Amber and CHARMM forcefields for ${n}_{\text{b}}$ = 30.

As noted previously, simulations of poly-dT and -dA30 performed with the Amber-bsc1 forcefield, generated structures did not depart from the initial half-helix configuration (Fig. S4)^21^. This outcome is in contradiction with the SAXS and smFRET experimental data that poly-dT is a somewhat disordered polymer chain, even under low salt conditions^21,64^. Overall, the simulations indicated very similar conformational properties for poly-dT and poly-dA as well as comparatively narrow ${R}_{\text{g}}$ vs $R$ distributions for both (Figs. S4, S5). This is reflected in the similarity of their corresponding calculated molecular effective charge values, $\left|{q}_{\text{calc}}\right|\approx$ 22.$6\pm$ 0.1 *e* (blue and red square symbols in Fig. 4C). This result is in obvious qualitative disagreement with our experimentally measured ${q}_{\text{eff}}$ values that displays clear differences of approximately 8% between poly-dT and poly-dA (open circles in Fig. 4C).

The CHARMM36 forcefield, on the other hand, appeared to perform much better at capturing the conformational properties of ssNAs in general. For instance, the results compare more favourably with the experimentally reported ${R}_{\text{g}}$ and $R$ values for poly-dT30, as well as with inferences on the ‘local’ polymer chain properties from SAXS measurements at 20 mM NaCl (see Supplementary Information Sect. S4 for further discussion)^15,23,43^. Interestingly, simulations with the CHARMM forcefield revealed a marked difference in conformational ensembles for poly-dT and poly-dA sequences, with the poly-dA structures characterised by collapsed conformations that resided in metastable hairpin structures, as reflected in a small average end-to-end distance (${R}_{\text{g}}\approx 2.3$ nm and $R\approx 3$ nm), well below the reported SAXS values of ${R}_{\text{g}}=2.72$ and $R=7$ nm for poly-dA30 (Fig. S5)^23^. However, such globular forms of poly-dA have not been observed in SAXS experiments up to salt concentrations as high as 1 M and possibly points to a shortcoming of the CHARMM36 forcefield in modelling poly-dA^28^. Indeed we found that the simulated conformations for poly-dA, and to a lesser extent for poly-dT, gave calculated values of molecular effective charge that fell significantly short of those measured by ET*e* by about 20% (blue and red triangle symbols in Fig. 4C). This is not surprising, given that conformational compactness generally entails higher charge renormalization ($\eta <$ 1) and therefore a lower magnitude of effective charge. Nonetheless, despite the fact that the absolute ${q}_{\text{calc}}$ values were lower by about 20% than the measured ${q}_{\text{eff}}$ values (e.g., poly-dT30 ${|q}_{\text{calc}}|\approx 21.5\, e$, $|{q}_{\text{eff}}|\approx 27.1\,e)$, the qualitative trend indicated by simulations that poly-dT has a less compact conformation than poly-dA ($\approx$ 7% disparity in ${|q}_{\text{calc}}|$) is indeed captured in experimental data that points to an $\approx$ 8% disparity in $|{q}_{\text{eff}}|$. However, for poly-rU RNA, the CHARMM36 forcefield resulted in more extended structures and thus higher magnitudes of ${q}_{\text{calc}}$ values than the DNA homopolymers (see Figs. 4, S5)—a trend clearly not observed in the ${q}_{\text{eff}}$ values measured by ET*e*. Such disparities between measurement and structural modelling trends was a recurring theme for the force-fields examined in this study, except for DES-Amber discussed next.

### The specialised DES-Amber and HB-CUFIX forcefields

Next we analysed results from DES-Amber, a next generation ssNA forcefield. We found that DES-Amber (star symbols in Fig. 4C) was able to better capture experimental sequence dependent trends in ${q}_{\text{eff}}$ for ssNAs, i.e., $\left|{q}_{\text{eff}}\right|$ of poly-dT >  -rU $\ge$ -dA. The DES-Amber forcefield overcomes some of the deficiencies seen with the Amber and CHARMM36 models, with the ssDNA exhibiting some degree of flexibility whilst not engaging in extensive internal base-pairing that results in the DNA collapsing into globular conformations. Furthermore, DES-Amber indicates minor differences in the conformational ensembles of poly-dT and poly-dA, with poly-dT exhibiting a higher degree of polymer flexibility (able to transiently access more extended and collapsed conformations) and poly-dA adopting more rigid and compact structures with a higher degree of inter-base stacking (Fig. 4B). These trends may be seen to be reflected in the ${|q}_{\text{calc}}|$ values, which are $\approx$ 22.8 $\pm$ 0.15 *e* and $\approx$ 22.6 $\pm$ 0.08 *e* for poly-dT and poly-dA respectively (Fig. 4C). Importantly however, this difference in effective charge between the two species of $\approx$ 0.2 *e* ($\approx$ 1%) remains substantially lower than the experimentally measured disparity in ${q}_{\text{eff}}$ of $\approx$ 2 *e* ($\approx$ 8%) measured by ET*e*. Such quantitative discrepancies between experimental readouts and DES-Amber modelling results are also evident in other studies. For example, whilst values of ${R}_{\text{g}}$ for both poly-dA and -dT modelled with DES-Amber are in good agreement with the reported experimental SAXS value of approximately $3$ nm, the DES Amber mean end-to-end length ($\approx$ 9 nm) for both cases appears to be larger than the experimentally reported values of $R\approx 7$ nm at the lowest salt concentrations probed^23,60^.

Finally we turn our attention to modelling results for poly-rU. The DES-Amber forcefield was introduced as being able to model RNA successfully, having been validated to capture the end-to-end length for RNA as measured by smFRET^65^. Importantly, we found that the DES-Amber forcefield generated structures for DNA and RNA whose calculated ${|q}_{\text{calc}}|$ values were indeed significantly different (see Fig. 4C). The MD conformational ensemble for DES-RNA reflects a higher degree of polymer flexibility and a much shorter average end-to-end length (${R}_{\text{g}}\approx$ 3 nm and $R\approx$ 8 nm) than its DNA counterpart (see Fig. 4B). Furthermore, the average contour length of DES-RNA (calculated as the sum of inter-phosphate distances along the polymer chain) was calculated to be 17.2 nm for ${n}_{\text{b}}=30$, considerably shorter than poly-dT, which measured 19.5 nm. This ratio in contour lengths of poly-rU to -dT is 0.88, which is in close agreement with the value of $\approx$ 0.87 reported using a combination of SAXS and smFRET in Ref.^24^, albeit for measurements performed under different conditions. We expect the suggested reduced spatial extent and inter-backbone charge spacing of the RNA polymer chain to result in a reduced molecular effective charge for RNA compared to DNA. Indeed, we found that representative configurations for 30 base poly-rU and -dT from DES-Amber have average ${|q}_{\text{calc}}|$ values of 22.1 and 22.8 *e* respectively, giving a ratio of 0.97 between -rU and -dT (Fig. 4C). Despite the fact that the absolute values are similar but not identical to the measured values (${|q}_{\text{eff}}|\approx$ 26.2 $\pm$ 0.12 *e* and 27.4 $\pm$ 0.1 *e*), the qualitative trend obtained for the simulated molecular structures, as reflected in the ratio of the two values, is in fact captured in the electrometry measurements that indicate a ratio of 0.96. Thus, experimentally measured disparities between the two molecular species are indeed successfully captured in DES-Amber simulations. It is worth noting again in this context, that whilst the ET*e* measurements are of high precision ($\le$ 1% in this work), which enables differences and relative trends in effective charge between molecular species to be measured reliably, the accuracy of the absolute values of ${q}_{\text{eff}}$ can display around 10% uncertainty in the present measurements which precludes rigorous testing against absolute ${q}_{\text{eff}}$ values for computed structures. Furthermore, minor additional uncertainties on modelling parameters enter the picture on the computational side, carrying implications for quantitative comparisons of absolute values between experiment and simulation. One source of uncertainty in the electrostatic modelling of molecular structures concerns the width of the ion accessible region, *w*, surrounding the molecular structure, which introduces a source of variation in the magnitude of the ${q}_{\text{calc}}$ values that may well account for about 5% of the presently noted disparity in absolute values. For instance, increasing the value of $w$ from 0.2 to 0.4 nm raises the magnitude of the ${q}_{\text{eff}}$ by $\approx$ 2% per Å for 30 and 60 base poly-dT. Currently therefore, these considerations in both experiments and computation taken together place constraints on the ability of our present methodology to clearly highlight shortcomings and gaps in molecular simulation models when disparities in absolute values of ${q}_{\text{eff}}$ between experiments and structural models lie in the range of $\approx$ 10% or less. Despite the noted disparities between absolute values of effective charge from measurement and computation, inferences based on measured differences or relative trends in effective charge between species measured under identical conditions are less subject to experimental inaccuracy and model-parameter uncertainty in computation. Comparative trends in effective charge therefore do nonetheless provide a suitable basis on which to compare experiments with computational indications, and may aid in the fine-tuning of molecular models.

Finally, we employed an alternative ssRNA model, HBCUFIX, which has been demonstrated to better capture the persistence length and ${R}_{\text{g}}$ of short poly-rU chains compared to DES-Amber^66^. We found that the conformational landscape sampled by this molecular model was overall similar to that of DES-Amber, exhibiting a substantial exploration of the conformational parameter space, reflecting that of a flexible polymer chain (see Fig. S5). The effective charge value of a representative poly-rU structure using the HB-CUFIX model of ssRNA gave ${|q}_{\text{calc}}|\approx$ 21.3 *e,* similar to the DES-Amber value of $\approx$ 22.3 *e*. Thus we found that under the conditions of this study, the specialised ssRNA forcefield descriptions generated effective charge values that were in good mutual agreement.

#### The coarse grained oxDNA model

Having examined and compared our experimental data with a range of atomistic MD models we then performed a comparison of the results of oxDNA2, a coarse-grained DNA model, with our experiments for both nucleic acids lengths ${n}_{\text{b}}=$ 30 and 60^67^. We proceeded along the same lines, relating computed effective charge values, ${|q}_{\text{calc}}|$, with experimental measurements as described previously. Since oxDNA was optimised to predict the self-assembly, structure and mechanical and thermodynamic properties of DNA nanostructures, e.g., DNA origami, it is not entirely clear if oxDNA predictions may generally be relied upon as providing an accurate picture of ssNA molecular 3D conformation^67^. However, the oxDNA2 model incorporates a simple combination of both sequence-specific inter-base stacking strengths and a salt dependent non-bonded interaction term between backbone sites that could be sufficient to describe the molecular configuration of DNA. In fact we found that computed trends in ${|q}_{\text{calc}}|$ values for oxDNA structures converted to all atom structures (see “Methods” for details) compared favourably with experimental ${|q}_{\text{eff}}|$ trends (i.e., $\left|{q}_{\text{eff}}\right|$ poly-dT > mixed sequence $>$ -dA) determined with ET*e* for DNA, for both ${n}_{\text{b}}$ = 30 and ${n}_{\text{b}}$ = 60 (crosses in Fig. 4C). For example, the ratios in ${|q}_{\text{calc}}|$ values of poly-dT to poly-dA, modelled with oxDNA, were calculated to be $\approx$ 1.03 and 1.06 for ${n}_{\text{b}}$ = 30 and ${n}_{\text{b}}$ = 60 respectively, which finds qualitative agreement with the ET*e* measured ratios of 1.08 and 1.11. At the same time, the mean end-to-end length, $R\approx 10$ nm, implied by the oxDNA results for poly-dT30 at the salt concentrations of our measurements (ca. 1 mM) is similar to DES-Amber ($R\approx$ 9 nm) but is slightly larger than SAXS measurements, implying some quantitative disparities in comparisons of oxDNA results with other experimental data^23,43^.

We note however, that although successful for DNA, the oxRNA model, which incorporates the weakest inter-base stacking between adjacent U bases compared to A and T, generated structures with the largest values of ${R}_{\text{g}}$ and $R$, and the largest ${|q}_{\text{calc}}|$ values of all sequences considered, similar to the results obtained with the CHARMM36 forcefield (see Fig. 4C). This trend, reflected in the ratio of ${|q}_{\text{calc}}|$ values for poly-dT to poly-rU of approximately 0.97, does not agree well with our measured ratio in ${|q}_{\text{eff}}|$ of 1.07 for ${n}_{\text{b}}=$ 60. Thus, of all the models tested for RNA it appears that the DES-Amber forcefield better captures the trend in effective charge measurements. This likely points to the importance of the incorporation of QM-level accurate base stacking and torsional energetics in determining RNA 3D conformation^65^.

In summary, ET*e* reveals a lower |${q}_{\text{eff}}|$ value for poly-dA DNA compared to poly-dT, which we may attribute to a more compact polymer structure by comparing our measured trends with those that are reflected in MD model structures. At the microscopic level, this compaction is believed to arise from an increased inter-base stacking strength of neighbouring adenine bases compared to thymine (see Fig. 4B), further experimental evidence for which can be found from AFM pulling measurements^68–70^. Many of the other inter-base stacking strengths (including G, C and other pairwise permutations) have been calculated to lie in between these two extrema which can in fact be seen to be reflected in the intermediate value of both ${q}_{\text{eff}}$ and electrophoretic mobility of the mixed sequence case for ${n}_{\text{b}}$ = 60 (see Figs. 4C, 3C)^71^. However, it is interesting that the ET*e* measurements place poly-rU as having an effective charge similar to that of poly-dA, whereas arguments on base stacking strength alone would place poly-rU as having the highest |${q}_{\text{eff}}|$ of all homopolymers examined in this study, as also suggested by coarse grained oxRNA simulations. However, poly-rU structures obtained with the DES-Amber forcefield clearly indicate a shorter average end-to-end distance $R$ and contour length ${l}_{\text{c}}$ for RNA compared to DNA, and capture the ET*e* readout better.

#### The rigid-rod model of ssDNA

Although molecular simulations are growing increasingly sophisticated and powerful, field theoretical descriptions remain important in quantitative modelling of experimental data. This is particularly important in descriptions of many-body phenomena where computational expense grows rapidly with system size. For example, in a solution phase electrostatics simulation a typical atomistic computation could involve at least 10^10^ ions and up to 1000 times as many water molecules.

Thus in order to glean further physical insight into the experimental trends observed for ${q}_{\text{eff}}$ as a function of ssNA sequence we sought to relate our measured effective charge values to simple rigid rod models of the ssNAs within a continuum electrostatics framework. Although for dsDNA the rigid rod electrostatic model is an excellent approximation of the atomic level reality, its appropriateness for describing the interactions of single stranded nucleic acids is less obvious^38,40,62^. Specifically, given the high conformational flexibility of the disordered polyelectrolyte chain, it is not clear that the charged groups in the molecule could be reasonably expected to lie on the surface of a rectilinear rod as suggested in the simplest models of polyelectrolyte theory^40,72,73^. Interestingly however, a rigid rod model of ssDNA has been shown to be sufficient in PB modelling of the ion excess as measured by AES experiments^62^.

**Fig. 5:**
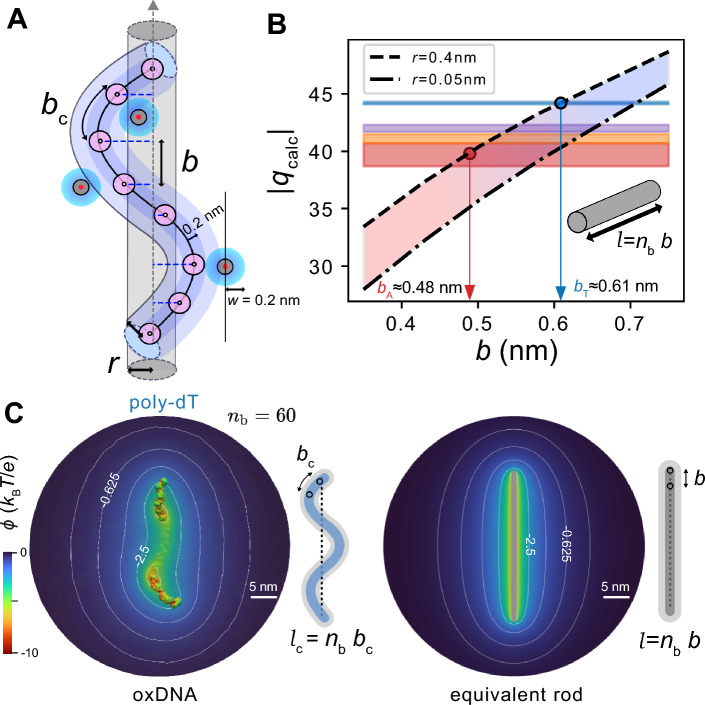
The rigid rod model for a single stranded nucleic acid. (**A**) Schematic depiction of a flexible ssNA backbone contour consisting of phosphate sites (pink spheres) occupying the center of a tube of finite diameter that represents the volume excluded to the center of mass of an ion. A further thickness of $w\approx$ 0.2 nm produces an ion accessible surface that successfully incorporates the role of the finite size of a hydrated cation. The equivalent rigid rod is depicted as a grey vertical cylinder with axial inter-charge spacing $b$. (**B**) Calculated values of effective charge ${|q}_{\text{calc}}|$ vs axial inter-charge spacing $b$ for the rigid rod models of radius $r=$ 0.05 and 0.4 nm and length $l={n}_{\text{b}}b$. ET*e* measurements for ${n}_{\text{b}}=$ 60 shown as horizontal coloured bands for sequences poly-dT (blue), -dA (red), mixed DNA sequence (purple) and -rU (orange). Inferred $b$ values are ${b}_{\text{T}}\approx 0.61-0.71$ nm, and ${b}_{\text{A}}\approx 0.48-0.59$ nm for poly-dT and -dA based on the present range of of $r$ values (downward pointing arrows). Linear fit equations to the data corresponding to $r=$ 0.05 and 0.4 nm in the ranges of interest are ${|q}_{\text{calc}}| =44.2 b+13.22$ and ${|q}_{\text{calc}}| =37.36 b+21.25$ respectively. (**C**) Electrostatic potential distribution obtained by solving the PB equation for a representative oxDNA poly-dT structure (${n}_{\text{b}}$= 60) converted into an all-atom model with a width parameter of $w=$ 0.2 nm (left). The polymer is characterised by an end-to-end distance $R=22.5$ nm and contour length ${l}_{c}=$ 36 nm. The effective charge of this polymer is matched by an equivalent rod model with dimensions $r$ = 0.4 nm and $b$ = 0.5 nm (right). Electrostatic potential contours are shown at $\phi =$ − 5, − 2.5, − 1.25, − 0.625 and − 0.3125 ${k}_{\text{B}}T/e$. The average electrical potential on the surfaces of the poly-dT structure and rod model are − 5.295 and − 6.58 ${k}_{\text{B}}T/e$ respectively.

We calculated the effective charge of uniformly charged rigid rods of radii $r$ = 0.05 and 0.4 nm using our continuum electrostatics calculation framework (see “Methods”). At the lower limit the rod radius approximates a line charge, whilst the upper radius limit may be thought to incorporate the excluded volume due to the finite size of the ssNA backbone atoms as well as that of a hydrated cation (Fig. 5A). We have previously shown that accounting for the excluded volume due to ions and water of hydration at the molecular interface in this manner permits us to incorporate the role of finite ion size in a point-ion based PB model^34^. Previous studies on the generation of molecular surfaces for PB calculations suggested that a value of $w\approx$ 0.2 nm produces an ion accessible surface for an atomistic structure that successfully incorporates the role of the finite size of a hydrated ion^34^. Here, $w$ is a parameter which represents a thickness to the molecular surface in addition to that represented by the vdW surface. Together with the radius of the backbone atoms of $\approx$ 0.2 nm we may therefore expect a rod of radius $r\approx$ 0.4 nm to provide a reasonable description of a ssNA molecule within the rod model. We calculated the effective charge, ${q}_{\text{calc}}$, for rods of length $l={n}_{\text{b}}b$ carrying a total charge of ${q}_{\text{str}}=-\left({n}_{\text{b}}+1\right) e$, where $b$ is the axial charge spacing (Fig. 5A,C). We focused on rod lengths corresponding to ${n}_{\text{b}}=60$ as this represents the most highly charge renormalizing regime in our experiments. An estimate of the renormalized charge of two ATTO dye molecules was added to the effective charge value calculated for a rod representing a label-free 60 base DNA in order to facilitate comparison with experiment (see “Methods” and Fig. S3). We determined ${q}_{\text{calc}}$ values for rods of various axial spacings $b$ ranging from 0.35 to 0.65 nm. Setting ${q}_{\text{calc}}={q}_{\text{eff}}$ in the obtained ${q}_{\text{calc}}$ vs $b$ relationships then yielded an estimate of the value of $b$ corresponding to each of the two values of rod radius for each molecular species (Fig. 5B).

At the upper limit of rod radius, $r=$ 0.4 nm, we obtained inferred values of $b=$ 0.61 nm for poly-dT and $b\approx$ 0.5 nm for poly-dA and poly-rU (Fig. 5B). These values are comparable with the values of contour length per base, ${b}_{\text{c}}$, obtained for poly-dT and poly-rU in SAXS experiments on 40 base ssNAs of about 0.6 and 0.5 nm respectively^24^. Specifically, SAXS measurements report ${b}_{\text{c}}=$ 0.56 nm and 0.49 nm for poly-dT and poly-rU respectively (see Table 1) and this trend is in good agreement with our inferences of axial charge spacing based on the rod model. However, we in fact expect a quantitative disparity between $b$ and ${b}_{\text{c}}$ since the rigid rod model effectively replaces a curvilinear chain representing a flexible polyelectrolyte by a rod with a single rectilinear axis and a projected axial charge spacing, $b$ (Fig. 5A). For a disordered single-stranded polynucleotide structure therefore, $b$ is to be interpreted as the mean charge spacing along the rectilinear axis which is expected to be smaller than ${b}_{\text{c}}$^72–74^ (Fig. 5A,C; see Supplementary Information Sect. S6 for further detail). The fact that our present results indicate ${b\approx b}_{\text{c}}$ may in fact point to a current overestimate of measured ${q}_{\text{eff}}$, as encountered and previously discussed in the comparison of the experimental ${q}_{\text{eff}}$ with ${q}_{\text{calc}}$ from MD models.

**Table 1:**
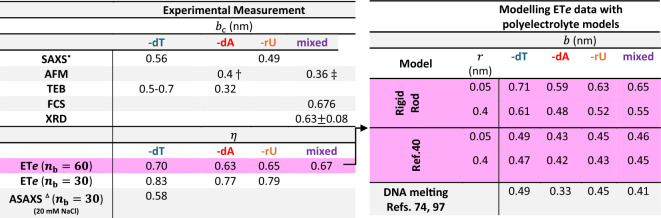
Left: tabulated values of the contour length per base, ${b}_{\text{c}}$, measured experimentally with techniques such as SAXS (*—Ref.^24^), AFM (†—Ref.^98^, ‡—Ref.^99^), transient electric birefringence (TEB, Ref.^100^), fluorescence correlation spectroscopy data in combination with a mean field theory (FCS, Ref.^75^) and from X-ray diffraction of ssDNA-protein complexes (XRD, Ref.^26^). ET*e* (and ASAXS in one case, Δ—Ref.^43^) measures a charge renormalization factor, $\eta$ (bottom rows); right: $\eta$ values for ${n}_{\text{b}}=60$, the most highly charge renormalizing regime, can be mapped onto a value of the axial base spacing, $b$, using various polyelectrolyte models, such as the rigid rod model discussed in the main text, or by means of a theoretical model such as that in Ref.^40^ (pink shaded rows).

Calculated values of $b$ inferred from studies of DNA melting data in combination with a charge renormalization model, such as in Refs.^74,97,^ are also presented for comparison.

For the simple line charge description ($r=$ 0.05 nm) in turn, we inferred $b$ values of 0.71, 0.65, 0.63 and 0.59 nm for poly-dT, mixed, -rU & -dA sequences respectively (Fig. 5B). These axial charge spacings are higher than most estimates of ${b}_{\text{c}}$ (see Table 1), but do appear to find agreement with some experimental values obtained for the contour length per base for short ssNA oligomers in ssNA-protein complexes as measured by X-ray diffraction (XRD, Ref.^26^), and inferred for long ssNAs from fluorescence correlation spectroscopy data (FCS, Ref.^75^). Thus it appears that the rod model may not only provide a meaningful description of the electrostatics, but in doing so appears to be able to quantitatively capture an indication of the interphosphate spacing along the backbone contour, reflecting the essential impact of charge density on the electrostatics problem for ssNAs. This simplified view of the problem may therefore furnish a relatively straightforward modelling framework within which to interpret and parametrise differences in local structure as reflected in charge spacing in disordered biomolecules in solution.

## Conclusion

We have measured effective charge values of single stranded nucleic acids of varying lengths and sequences in solution using molecular electrometry. We find that the magnitude of effective charge of poly-dT oligonucleotides is significantly higher than poly-dA and poly-rU sequences, despite all species carrying identical structural charge. These measurements indicate that conformational and structural differences between ssNAs of varying sequences are strongly reflected in their electrostatic properties. Since ET*e* measurements are performed under thermal and mechanical equilibrium in solution, and in the absence of external fields and sieving matrices, the measurements are amenable to computational modelling using both coarse grained and atomistic approaches. Comparing experimental data with calculated effective charge values determined using all-atom and coarse-grained simulations of ssNAs suggests that the lower measured effective charge values of poly-dA, as compared to poly-dT, arises from more compact molecular conformations, brought about arguably by enhanced inter-base stacking energies for adenine bases compared to thymine. For poly-rU oligonucleotides, structural modelling using a specialised ssRNA forcefield points towards a shorter average contour length than DNA and increased flexibility of the polymer chain, which in turn seem to underpin the consistently lower magnitude of both measured and calculated effective charge values for poly-rU compared to poly-dT. Our comparison of measured values of effective charge for ssNAs with those calculated for the same species using a variety of molecular models has highlighted some deficiencies of popular all-atom forcefields, thus recapitulating the widely recognised need for improved forcefield descriptions of nucleic acids^63^. Although our measurements of effective charge for the longer oligomers agree with the general magnitude of the corresponding computed values, we note at present, a consistent overestimate of 10–20% of measured values compared to computed quantities. Given that the present level of inaccuracy of ET*e* is expected to be around 10%, the present results would still suggest a residual disparity whose origin may become clearer with advances in modelling procedures or indeed further experimentation. We have further found that mapping our measured effective charge values for ssNAs on to that of a uniformly charged rod not only captures the essential impact of charge density on the electrostatic problem but also, importantly, yields estimates of a key structural parameter—the average contour length per base, ${b}_{\text{c}}$—describing the molecule, which we find are in good agreement with measured values using SAXS and X-ray diffraction.

Finally, ET*e* offers significant operational advantages over state-of the-art structural methods such as AES and SAXS in that the measurement is performed using a standard wide-field fluorescence microscope on sub-zeptomole amounts of sample, e.g., 100 molecules yields a measurement precision on ${q}_{\text{eff}}$ smaller than 3%, and in a typical measurement duration of 1 min which can be significantly improved upon by measuring a larger number of molecules or for longer durations. Furthermore, the small measurement imprecision (< 1%) readily attainable in ET*e* permits small differences in charge renormalization between species to be measured with high certainty, which may not hold for ion counting experimental readouts (see Supplementary Information Sect. S7). Importantly, similar to FRET, the shorter measurement durations characteristic of single molecule optical detection experiments, and the access to individual molecular behaviour in real time, offers the prospect of detecting molecular conformation dynamics—albeit on the sub-second timescales—that is not readily accessible via other approaches. ET*e* therefore combines high measurement precision with several advantages of existing experimental methods directed at reading out molecular conformational properties. Finally, although the ET*e* measurements in this work were performed in low salt conditions (approximately 1 mM NaCl), further improvements to the methodology and device design should in future enable measurements at higher ionic strengths of 50–100 mM. As shown in Figs. 1 and S2, the depth of the potential well depends on the electrical potential at the midplane of the slit, ${\phi }_{\text{m}}=2{\phi }_{\text{s}}\text{exp}(-\kappa h)$, which suggests that reduction of the slit height to the range of $h=$ 10–20 nm will support measurements at ionic strengths of around 50 mM. Divalent or multivalent cations may further be present in the electrolyte noting that they contribute more strongly to the total ionic strength than monovalent salts. Future improvements may also entail label-free imaging of the escape dynamics and molecular property measurements in the solution phase^76^.

ET*e* thus offers a facile and rapid measurement approach that opens up the dimension of effective charge as a means to test and validate outputs of theoretical and computational platforms for structural and conformational modelling of charged disordered biomolecules. The ability to detect subtle differences in 3D conformation of nucleic acids has immediate ramifications for the rapid, high throughput, coarse-grained characterization of biomolecular structures and conformation in the solution phase. This combined approach will likely foster structural and conformational inferences and modelling of more complex molecules such as folded RNAs for which structural studies lag significantly behind their protein counterparts^77^. Beyond examining the conformation of molecular species in the pure state, the approach could also provide a platform to screen and investigate molecular conformational changes induced by the binding of small-molecule ligands and metabolites to proteins and riboswitches, a process which has far reaching implications in gene expression, enzymatic activity and drug design^78,79^.

## Methods

### Experimental methods

#### Characterization of DNA and RNA samples

The short chain nucleic acid fragments and ${n}_{\text{b}}=$ 60 ssDNA fragments were purchased from Microsynth. ${n}_{\text{b}}=$ 30 dsDNA and ${n}_{\text{b}}=$ 60 ssRNA samples were purchased from IBA Lifesciences (Germany) and all ${n}_{\text{b}}=$ 30 ssNA samples were purchased from Integrated DNA Technologies (IDT). All fragments were doubly labelled with ATTO 532 dye molecules, apart from the ${n}_{\text{b}}=$ 5 ssDNA sample which was singly labelled (see Fig. S1 for a chemical diagram). The ssNA samples were examined with 20% polyacrylamide native gel electrophoresis (Novex™ TBE Gels, 20%, ThermoFisher Scientific) (Fig. 3C). Sequences for the short chain DNA fragments are as follows: ${n}_{\text{b}}=$ 12: TAG AAC TAG TGG, ${n}_{\text{b}}=$ 11: TAG AAC TAG TG, ${n}_{\text{b}}=$ 9: TAG AAC TAG, ${n}_{\text{b}}=$ 8: TAG AAC TA ${n}_{\text{b}}=$ 5: TAG AA.

#### Measuring the effective charge of single stranded nucleic acids (ssNAs) using Escape Time Electrometry (ET*e*)

Devices for ET*e* measurements were fabricated to the same specification and using procedures described previously^32–34,36,37,53^. Landscapes of electrostatic traps were created in nanoslits of height $2h=$ 70–75 nm and channel width $=$ 5 $\upmu$m, with nanostructure pocket depths $d=$ 280 nm, and radius $=$ 300 nm (see Fig. 1A). The height of the nanoslits were measured using atomic force microscopy. Nanoslits were loaded with a suspension of the molecular species at a concentration of 100–125 pM. Solution pH and conductivity was measured before and after the measurement using a micro-pH electrode (SevenDirectSD23, Mettler Toledo, UK) and microconductivity meter (Laquatwin, Horiba Scientific, Japan). ssNAs were loaded into an ET*e* device which consists of parallel nanoslits each containing arrays of electrostatic fluidic traps as shown in Fig. 1.

Wide-field fluorescence microscopy of the escape dynamics of molecules in the trap landscape was performed using excitation from a 532 nm DPSS laser (DMPV-532-3, Del Mar Photonics) that was focused at the back aperture of a 60 $\times$, NA = 1.2 water immersion objective (Olympus, UK). Time lapse videos were recorded at 10–20 Hz using an exposure time ${t}_{\text{exp}}=$ 5 ms, for a measurement time of 3–5 min using a sCMOS camera (Kinetix Photometrix). Extensive details of the ET*e* measurement technique are provided in previous work, contained in Refs.^32,36,37,53^. As in previous work, the probability density function of residence times of molecules in the traps, $P\left(\Delta t\right)\propto \left(\frac{1}{{t}_{\text{esc}}}\right)\text{exp}\left(-\frac{\Delta t}{{t}_{\text{esc}}}\right)$, was recorded and used to extract the average molecular escape time, ${t}_{\text{esc}}$. In general we do not include in the fitting process 1–2 data points at the shortest measured lag times, $\Delta t$. This is because escape events recorded at the shortest durations can contain contributions from transiently trapped, weakly charged molecular species such as free dye, degraded material, or free ssDNA strands in the background solution in the case of the dsDNA calibrator molecules. The average escape time is related to the well depth $W$ according to Kramer’s relation ${t}_{\text{esc}}\propto \text{exp}\left(\frac{W}{{k}_{\text{B}}T}\right)$^80^.

In order to convert measurements of ${t}_{\text{esc}}$ to the depth of the trap, $W$, we performed Brownian dynamics (BD) simulations of the escape process. Full details of BD simulations are covered extensively in our previous works, particularly in Refs.^32,36,37,53^. In our BD simulations, we treat molecules as effective spheres of a radius equal to the measured hydrodynamic radius, ${r}_{\text{H}}$, of the molecule, which was measured using fluorescence correlation spectroscopy. Observation of a large number of escape events ($N\approx {10}^{4}$) allows $W$ to be determined with a statistical uncertainty of about 1%^37^. Widths of presented Gaussian distributions of ${t}_{\text{esc}}$ therefore arise predominantly from the statistical error on the fitted ${t}_{\text{esc}}$ values which is approximately ${t}_{\text{esc}}/\sqrt{N}$. The total free energy $W$ can be written as the sum of the electrostatic free energy of interaction $\Delta {F}_{\text{el}}$ and an entropic term arising from the spatial fluctuations of the molecule $\Delta {F}_{\text{trans}}$. A substantial contribution to $W$ results from the electrostatic interaction free energy, ${\Delta F}_{\text{el}}={{F}_{\text{slit}}-F}_{\text{pocket}} ,$ which is the difference in the axial minimum of electrostatic free energies for a molecule residing in the “slit” and “pocket” states, as shown in Figs. 1A and S2^32,36,37,53^. $\Delta {F}_{\text{el}}$ may also be calculated for a given molecular structure as described in a subsequent section.

We have previously shown that effective charge values, ${q}_{\text{eff}}$ can be deduced via the relation $\Delta {F}_{\text{el}}={q}_{\text{eff}}\Delta {\phi }_{\text{mid}}$^35^. Here, ${\phi }_{\text{mid}}$ is the electric potential at the midplane of the nanoslit in the experimental device and is given by ${\phi }_{\text{mid}}\approx {\phi }_{\text{m}}\approx 2{\phi }_{\text{s}}$ exp($-\kappa h$) where ${\phi }_{\text{s}}$ is the effective surface potential at the slit walls, whereas in the pocket region, ${\phi }_{\text{mid}}$ is zero by design. In order to accurately measure the effective charge ${q}_{\text{eff}}$ for a molecule of interest we require knowledge of the surface potential ${\phi }_{\text{s}}$ of the ET*e* measurement device which is a priori unknown. Therefore, in order to determine ${\phi }_{\text{s}}$, a calibration measurement was first performed using a molecule of well-known effective charge, namely a 30 bp or 60 bp dsDNA molecule carrying two attached ATTO-532 dyes (${|q}_{\text{eff}}|=$ 28.2 and 45.6 *e* respectively, in this study). The effective charge values of the calibrator molecules were determined from previous work. Specifically, Ref.^34^ showed that the electrostatic properties of the double helix could be well described by a cylinder of radius $r=$ 1.3 nm (including that of a hydrated Na^+^ cation) and a rise per basepair of $b=$ 0.31 nm. The imprecisions in the measured values of ${t}_{\text{esc}}$ and ${q}_{\text{eff}}$ are $\sim$ 0.5 and $\sim$ 1.5% for the 30 and 60 bp calibrator molecules respectively, $\sim$ 0.5% for 30 base ssDNA, and $\sim$ 1% for 60 base ssDNA, which are suitable for capturing qualitative trends and differences arising from disparities in conformation between molecular species that lie in the range of $\approx$ 5–10% in this study.

### Simulation methods

#### Simulation protocol

This section discusses in detail the methods and procedures used to carry out the MD simulations in this work. Simulation input files, trajectories, and code for the analysis of the simulations performed in this study are available on our Figshare repository: 10.6084/m9.figshare.25392787.

Initial nucleic acid structures were generated with the Nuleic Acid Builder (NAB) as part of the AmberTools software^81^. These were then solvated via the CHARMM-GUI solution builder platform in a truncated octahedral box, leaving at least 1 nm of space between the molecule and the edge of the box^82^. The net charge of the molecule was neutralised with the corresponding number of monovalent Na^+^ counterions, and no additional background electrolyte concentration added so as to best approximate the added salt concentration of $\approx$ 1 mM NaCl in the experiments.

MD simulations were performed with GROMACS software^83^. The Lennard–Jones and short-range electrostatic interactions were calculated with cutoff values of 1.2 nm and 0.9 nm for simulations employing the CHARMM36 and the AMBER or AMBER variant (DES-Amber/HB-CUFIX) forcefields respectively. The particle mesh Ewald (PME) method was used to evaluate the long-range electrostatic interactions with 3D periodic boundaries and employed a 0.12 nm grid spacing. Constraints were applied with the LINCS algorithm to all bonds involving hydrogen atoms in the system. After energy minimization with the steepest descents algorithm, a short 1 ns equilibration was performed in the NVT ensemble at 100 K using a v-rescale thermostat and a timestep of 2 fs, followed by an equilibration in the NPT ensemble (1 atm, 300 K, 2 fs timestep) using a Berendsen barostat with a coupling constant of 1 ps—all steps with position restraints of 500 kJ mol^–1^ nm^–2^ on the heavy atoms of the molecule. In another 20 ns NPT equilibration (1 atm, 300 K, 2 fs timestep) using the Parrinello-Rahman barostat, these restraints were then gradually released before production simulations of at least 1 $\upmu$s duration (see Table 2). For those models which displayed greater exploration of conformational parameter space we ran longer simulations of up to 3 $\upmu$s (see Table 2). The first 100 ns of the production simulation trajectories were discarded in the subsequent analyses. We point out that although the required timescales needed for the full simulation convergence of flexible ssNAs may indeed be much larger than those considered here, we found that extending simulations beyond 1 $\upmu$s did not appreciably alter the conformational (${R}_{\text{g}},R)$ landscapes and therefore the results of this study.

**Table 2:** Summary of the MD simulations performed in this work.

Force field + water model	System	Duration (μs)
Amberbsc1 + TIP3P	dT30, dA30	1
Charmm36 + TIP3P	dT30	2
Charmm36 + TIP3P	dA30, rU30	1
DES-AmberSF1.0 + TIP4D	dT30, dA30	1
DES-Amber_RNA + TIP4D	rU30	3
HB-CUFIX + TIP3P	rU30	2
DES-AmberSF1.0 + TIP4D	${n}_{\text{b}}=$ 5–12 (short fragments)	0.5

#### oxDNA simulations

We simulated ssNAs at 298 K, in 1 mM salt, using oxDNA version 2 following a protocol outlined in Ref.^84^. We performed two independent VMMC simulations for each system with 2 × 10^7^ attempted steps per particle. The first 10^5^ moves were discarded in the subsequent analysis. Sequence dependent stacking strengths and hydrogen bond parameters from oxDNA2 were implemented. Representative structures taken from oxDNA trajectories, chosen using the same method as described below for the all-atom MD trajectories, were then converted to all atom PDB format with tacoxDNA and a short energy minimization was performed with the amberbsc1 forcefield^85^.

#### Poisson Boltzmann (PB) free energy calculations to calculate theoretical molecular effective charge, ${q}_{\text{calc}}$

The calculation of ${q}_{\text{eff}}$ has robust theoretical underpinnings in Poisson-Boltzmann theory for solution phase electrostatics which provides a satisfactory theoretical description of the experiments in the regime of low monovalent salt concentrations^35,38,54^. Model structures of ssNAs used in PB calculations were obtained from MD simulations as described in the main text. Atomic coordinates of the molecule in PDB format were converted into PQR format (containing atomic charge information) with the MDAnalysis package^86^. However, for all atomic forcefields considered in this study, a polymer chain with number of bases ${n}_{\text{b}}$ is characterised by a structural charge of ${q}_{\text{str}} = -\left({n}_{\text{b}}-1\right)$ *e*, due to the non-integer charges of the terminal residues (which for instance contribute $\approx$ − 0.7 and − 0.3 *e* respectively in the Amber formalism, rather than a total charge of − 1 *e* characteristic of the interior bases). Prior to performing a PB calculation on the model structure, we therefore modified the atomic partial charges of the hydrogen termini atoms (named H5T, H3T) by − 1 *e* each to give a model with charge $-({n}_{\text{b}}+1)$ *e*. This slight modification ensures that the model structures of the polymer chain have the same structural charge as the ‘nucleic acid core’ of the molecules in experiments (see Fig. S1 for a chemical diagram). In practice the molecules also carry covalently linked dye molecules that contribute to both the molecule’s total structural charge (${q}_{\text{str}}$ $=-({n}_{\text{b}}+3)$ *e*) as well its effective charge. The renormalized charge of the effective of the attached ATTO dye molecules, ${q}_{\text{eff},\text{dye}}$, (the value of which was itself found to depend on ${n}_{\text{b}},$ see Fig. S3) was added to the calculated molecular effective charge in order to facilitate comparison of ${q}_{\text{calc}}$ with the experimentally measured ${q}_{\text{eff}}$ values. Atomic charges for the model of an ATTO dye molecule were taken from the AMBER-DYES library^87^. Note that these calculated values for ${q}_{\text{calc},\text{dye}}=$ − 0.62 *e* (for ${n}_{\text{b}}$ = 60) are in excellent agreement with the previously measured values under the same experimental conditions^34^.

In order to calculate effective charge values for a given molecular structure we consider molecular representations based on an ion accessible surface (IAS) deduced from atomistic structures. Previous work inferring structural parameters for dsDNA and dsRNA using measured effective charge values from ET*e* in combination with PB molecular electrostatic models pointed towards the presence of a thin region around the nucleic acids that is inaccessible to point ions in solution. The thickness of this layer, $w$, was found to correlate with values for the hydrated cation radii^34,88^. In previous work, inclusion of an additional thickness in addition to the van der Waals (vdW) surface, $0.14\le w\le 0.3$ nm, permitted measured electrostatic free energies to be captured using a PB view based on point-ions in solution that does not explicitly consider finite ion size. Previous work suggested a value of $w\approx$ 0.2 nm could capture the IAS for experiments with Na^+^ ions in solution^88^. Therefore, for the molecular electrostatic models considered in this study, we assigned each atom (excluding hydrogen atoms) a radius equal to the vdW radius (taken from the MD forcefield), and added a further 0.2 nm to generate an effective ion accessible surface. We used the Nanoshaper package in order to generate molecular surfaces of atomic coordinates using a probe size of 0.1 nm^38,89^. These surfaces were subsequently processed with the GMSH package to generate the required 3D mesh for our finite-element calculation^90^.

We then proceeded to calculate theoretical ${q}_{\text{eff}}$ values of model ssNA structures in our PB interaction free energy framework, as previously outlined extensively in Refs.^33–35,38^. The governing equations were solved for the cylinder models and model molecular surfaces using the COMSOL Multiphysics and FEniCS finite-element software packages respectively^91–93^. We solved the non-linear PB equation for the outer region of the molecular surface (i.e. in the electrolyte) for both the ‘slit’ and ‘pocket’ states in the experimental geometry, as shown in Figs. 1A and S2. The principal axis of the molecule was aligned parallel to the slit in the $xy$ plane (i.e., this configuration represents the local minimum in free energy). In the electrolyte region, the electric potential, $\phi$, is given by the solution of the PB equation: $-\nabla \cdot \left(\epsilon {\epsilon }_{0}\nabla \phi \right)={\rho }_{e}$ , where $\epsilon {\epsilon }_{0}$ is the permittivity of the medium and, ${\rho }_{e}={\Sigma }_{i}{c}_{i}{N}_\textup{A}e{z}_{i}\text{exp}(\frac{-{z}_{i}e\phi }{{k}_{\text{B}}T})$ is the ion density in solution. Here ${c}_{i}$ denotes the bulk concentration of ions of species $i$ and is simply the salt concentration, ${c}_{0}$, in solution for monovalent salts used in these experiments.

We uniformly distributed the partial charges of each atom over spherical seeds of radius 0.2 Å which is substantially smaller than typical values of the vdW radii of atoms. It should be noted that although the absolute electrostatic energy of the interior depends on the radius of the spherical seeds, which is an arbitrary parameter, the free energy difference $,\Delta F$, is independent of this parameter due to a cancellation effect which arises from subtraction of electrostatic free energies in the pocket and slit, ${\Delta F}_{\text{el}}={F}_{\text{slit}}-{F}_{\text{pocket}}$ (Fig. S2). In practice, the contribution of the interior region of the atoms to the free energy difference and hence the effective charge is negligible^38^. Therefore, in our free energy calculation, we only consider the contributions from the solvent region. Accordingly, the free energy functional is given by ${F}_{\text{el}}={\int }_{V}[\frac{\epsilon {\epsilon }_{0}}{2}{\varvec{E}}.{\varvec{E}}-2{c}_{0}{N}_{\text{A}}{k}_{\text{B}}T(\text{cosh}\psi -\psi \text{sinh}\psi -1)]dV$ where $V$ is the volume of the solvent region, ${\varvec{E}}=-\nabla \phi$ is the electric field and $\psi =e\phi /{k}_{\text{B}}T$ is the non-dimensional electric potential^54,94^.

For cylinders the PB equation was solved using Neumann boundary conditions that specify constant charge densities at the surface of the cylinder models. A constant charge approximation of ssNAs holds due to the acidic nature of the phosphate groups under the conditions of the ET*e* experiment. The silica walls were also treated with constant charge boundary conditions, as discussed previously^34^. For the silica surfaces, we have ${-\epsilon }_{0}\epsilon \nabla \phi .\mathbf{n}={\sigma }_{\text{w}}$, where ${\sigma }_{\text{w}}=-0.1\, e/$nm^2^ is a nominal surface charge density and $\mathbf{n}$ is the normal vector pointing into the electrolyte.

The following matching boundary conditions were also satisfied at the molecular surface: ${{\epsilon }_{0}\epsilon }_{\text{mol}}\nabla {\phi }_{\text{mol}}.\mathbf{n}={\epsilon }_{0}\epsilon \nabla \phi .\mathbf{n}$ and $\mathbf{n}\times \left(\nabla \phi -\nabla {\phi }_{\text{mol}}\right)=0.$ Here, $\mathbf{n}$ is the normal vector pointing into the electrolyte and the above matching conditions imply the continuity of the normal component of the displacement field, $\varvec{D}=-\epsilon_{0}\epsilon \nabla \phi$, and the tangential component of ${\varvec{E}}$ across the interface, respectively. We then deduced the effective charge of the molecule from the relationship ${\Delta F}_{\text{el}}={q}_{\text{calc}}{\phi }_{\text{m}}$ where ${\Delta F}_{\text{el}}={{F}_{\text{slit}}-F}_{\text{pocket}}$ and ${\phi }_{\text{m}}$ is the electrical potential at the midplane of the slit in the absence of the molecule^35,54^.

#### Electrostatic visualization of MD structures

Solving the PB equation determines the electric potential on a 3D finite element mesh inside and outside of the molecular surface which contains the partial charges of atoms. We first labelled each point in the mesh according to whether it resided in either the molecular volume or the electrolyte region. The molecular volume was defined by generating a molecular surface from the atomic PQR file using the Nanoshaper package using a probe size of 0.1 nm as mentioned previously^38,89^. The electrical potential on the molecular surface can be obtained by interpolation of data from the 3D mesh onto the 2D surface. The process of interpolation and visualization of the data can be efficiently handled by Paraview which provides a wide range of filters for processing large datasets^95^.

## Supplementary Information

Supplementary Information.

## Data Availability

The datasets generated during and/or analysed during the current study are available in the ‘ssNA electrometry and molecular simulations’ repository at 10.6084/m9.figshare.25392787.
