# Supplementary material for: Sensing the structural and conformational properties of single-stranded nucleic acids using electrometry and molecular simulations
Source: Sci Rep. 2024 Sep 4;14:20582. doi: 10.1038/s41598-024-70641-x (PMC11375218; doi:10.1038/s41598-024-70641-x)
Supplement: Supplementary file 1 — Supplementary Information. [file 41598_2024_70641_MOESM1_ESM.pdf]

## Supplementary Information

# Sensing the structural and conformational properties of single-stranded nucleic acids using electrometry and molecular simulations

**Rowan Walker-Gibbons[1] †, Xin Zhu[1] †, Ali Behjatian[1], Timothy J. D. Bennett[1] & Madhavi Krishnan[1,2]***

† these authors contributed equally

* corresponding author

Corresponding Author information: Madhavi Krishnan

**Email:** madhavi.krishnan@chem.ox.ac.uk

## Supplementary Information

### S1 Experimental sample characterization

In this work, all ssNA samples were doubly labelled with two fluorescent ATTO-532 dye molecules, apart from the $n_{\mathrm{b}}$=5 oligomer which contained only a single label (see Fig. S1 for a chemical diagram). A doubly labelled ssNA oligomer containing a number of nucleobases $n_{\mathrm{b}}$ is hence characterized by a structural charge $|q_{\mathrm{str}}| = (n_{\mathrm{b}}+3)$ $e$, which results from the charges of $n_{\mathrm{b}}$+1 phosphates and the two ATTO dyes, which carry a net structural charge of $q_{\mathrm{str}} = -1$ $e$.

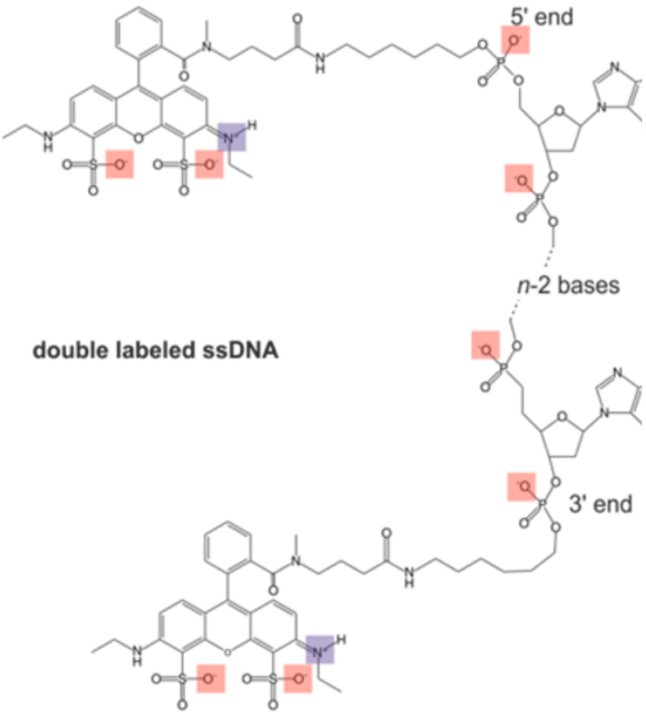


**Fig. S1. Chemical structure of experimental ssNA samples.**

## S2. PB calculation geometry and boundary conditions

As outlined in the main text and the Methods, we calculate theoretical effective charge values, $q_{\mathrm{calc}}$, of model structures in a Poisson Boltzmann framework that resembles the experimental ET*e* geometry. In Fig. S2 below we provide schematic representations of this geometry and highlight the governing equations and boundary conditions in the relevant domains.

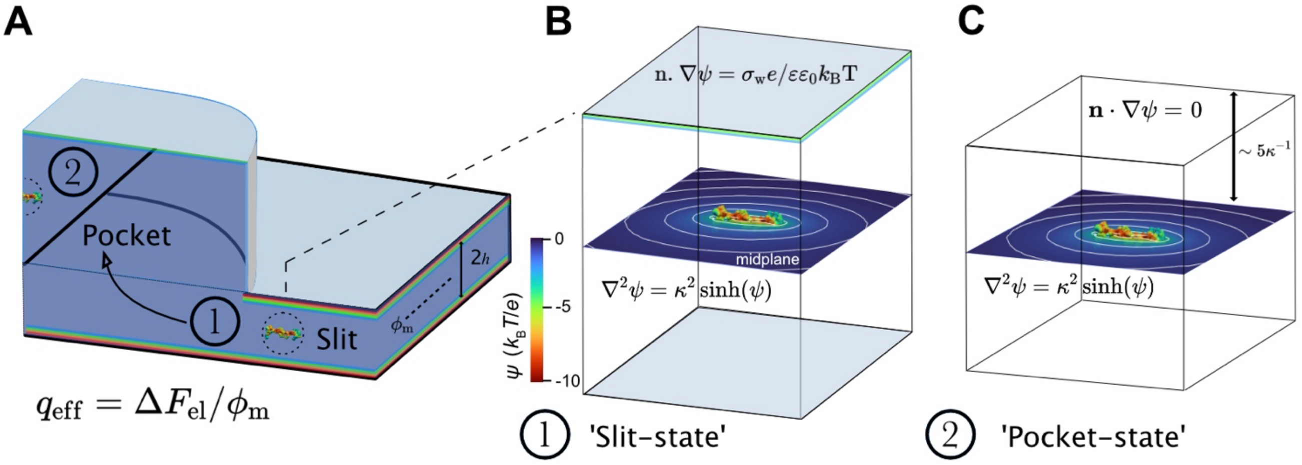


**Fig. S2. Schematic representations of model ssNA structures in PB free energy calculations.** (**A**) Illustration of the experimental ET*e* geometry, whereby molecules can either reside in 'slit' (1) or 'pocket' (2) states. The effective charge of the molecule can be deduced from the relationship $\Delta F_{\mathrm{el}} = q_{\mathrm{eff}}\phi_{\mathrm{m}}$ where $\Delta F_{\mathrm{el}} = F_{\mathrm{slit}} - F_{\mathrm{pocket}}$ and $\phi_{\mathrm{m}}$ is the electrostatic potential at the midplane of the slit. Electrostatic potential distributions for a molecule in the 'slit state' (**B**), and (**C**) the 'pocket state', i.e., effectively in free solution. The governing PB equation in the electrolyte and constant surface charge boundary conditions of the silica walls are depicted. The distance between the center of the molecule and the edges of the box was set to large distance of $5\kappa^{-1}$ as depicted in order to enable the implementation the zero electric field boundary condition on the outer walls of the geometry.

**S3. Determining the renormalized charge for a covalently attached ATTO532 dye molecule**

As discussed in the main text and the Methods, since our experimental structures contain attached fluorescent ATTO-532 dyes (which themselves carry a total structural charge $q_{\mathrm{str}} = -1\ e$), the contribution of the dye molecule must be added to the effective charge of model structures that do not include any dyes in order to generate, $q_{\mathrm{calc}}$ values, that can be compared with experimental measurements. Since an MD model for ATTO-532 was not available in the AMBER-dyes library and the dye is structurally very similar to ATTO-488, we performed a study calculating the effective charge of model half-helix structures with and without an attached ATTO-488-dye molecule [1]. The difference in the effective charge of these two structures yielded an estimate the renormalized charge of the ATTO-dye molecule, $q_{\mathrm{calc,dye}}$. Repeating this procedure for various values of $n_{\mathrm{b}}$ we found a dependence of this $q_{\mathrm{calc,dye}}$ on the number of bases, $n_{\mathrm{b}}$, of the attached polymer chain (see Fig. S3). The results are in line with the expectation of $\eta \to 1$ for small values of $n_{\mathrm{b}}$ and $\eta < 1$ for larger $n_{\mathrm{b}}$. In particular for large $n_{\mathrm{b}}$, we have $q_{\mathrm{calc,dye}}$= -0.62 *e* which is very close to the value of -0.46 *e* obtained for dsNAs in previous work [2].

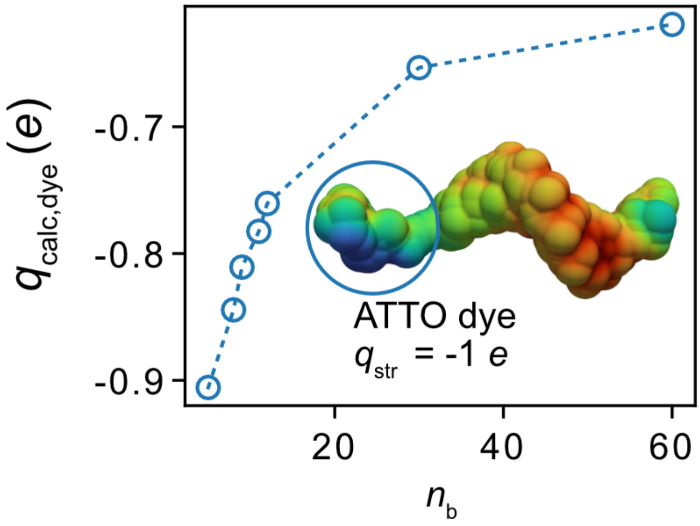


**Fig. S3: Renormalisation of the charge of an ATTO dye molecule as a function of the attached ssNA fragment length.** Values of the effective charge of an ATTO dye, $q_{\mathrm{eff,dye}}$, coupled to model 'half-helix' ssDNA structures of varying number of bases $n_{\mathrm{b}}$. $q_{\mathrm{eff,dye}}$ is determined as the difference in calculated effective charge of structures with and without an attached dye. The value of $q_{\mathrm{eff,dye}}$ for a given $n_{\mathrm{b}}$ is added to the effective charge of dye-free model ssNA structures to obtain a value that can be compared to the experimental measurements (see Methods).

**S4. Conformational analysis of model ssNAs**

In this section we describe further conformational analyses of model ssNA structures obtained using both atomistic and coarse-grained modelling approaches, and provide some discussion comparing the results with those inferred from SAXS data in the experimental literature. We also present conformational $R_g$ vs $R$ landscapes for all other ssNA models used in this work.

S4.1. Orientational correlation analysis

It has been demonstrated in Refs. 3,4 that the properties of ssNA polymer chains may be inferred from experimental SAXS data via a 'model building and iterative refinement' scheme that seeks to produce model ssNA structures that theoretically reproduce the measured SAXS profiles. One such property that can be inferred in this way is the directional persistence of the polymer chain, as captured by the orientational correlation function (OCF). The OCF is defined as $\langle \hat{r}_i . \hat{r}_j \rangle$, where $(\hat{r}_i . \hat{r}_j)$ are two phosphate site bond vectors in the ssNA chain, separated by $n$ bonds and where the dot product between two such vectors is computed as a function of the number of bonds that separate them ($|i - j|$) (see Fig. S4). The OCF can also be readily calculated as an output from analysing the molecular simulation trajectories in this work, and comparison with the experimentally inferred OCF data in Refs. 3,4 may provide an extra measure with which to validate ssNA structural models. We note that the experimental salt concentration for the SAXS measurements in Refs. 3,4 was 20 mM NaCl, close to our measurement salt concentration of $\approx 1$ mM.

The calculated OCF data for poly-dT and poly-dA modelled with the AMBER forcefield in this study showed very little difference between the two sequences, with both exhibiting a strong oscillatory motif which can be assigned to a highly stacked helical polymer chain configuration (see Fig. S4). Such poor agreement in the OCF profiles between the experimentally inferred and simulated data for the AMBER forcefields suggest that the models greatly over-estimate the tendency of nucleobases to form stacked helical coils. We found that the CHARMM-36 forcefield performed better than AMBER in capturing the OCF profiles for poly-dT and poly-dA (see Fig. S4). A clear difference in the orientational correlation function (OCF) between dT and dA for the CHARMM models was observed, with that of dA exhibiting a slight oscillatory motif, and with dT exhibiting a more gradual decay - characteristics also present in the experimentally inferred OCF profiles.

Recently, efforts to improve existing molecular dynamics forcefields for the purpose of modelling ssNAs have been made, as exhibited in the DES-Amber and CUFIX forcefields [5-9]. The combination of conformational constraints, hydrogen bonding, base stacking, salt and solvation effects that influence ssNA structure in an all atom MD model are challenging to accurately incorporate, and in reality no forcefield is able to closely match the experimental data across the wide range of solution conditions seen in experiments [10]. For DES-Amber poly-

dT we noticed a strong tendency for thymine bases to also form stacked helical regions, similar to the AMBER forcefield on which the model is based, resulting in an oscillatory motif in the OCF (see Fig. S4). However this behaviour is not inferred for poly-dT from the experimental SAXS data as previously discussed [3].

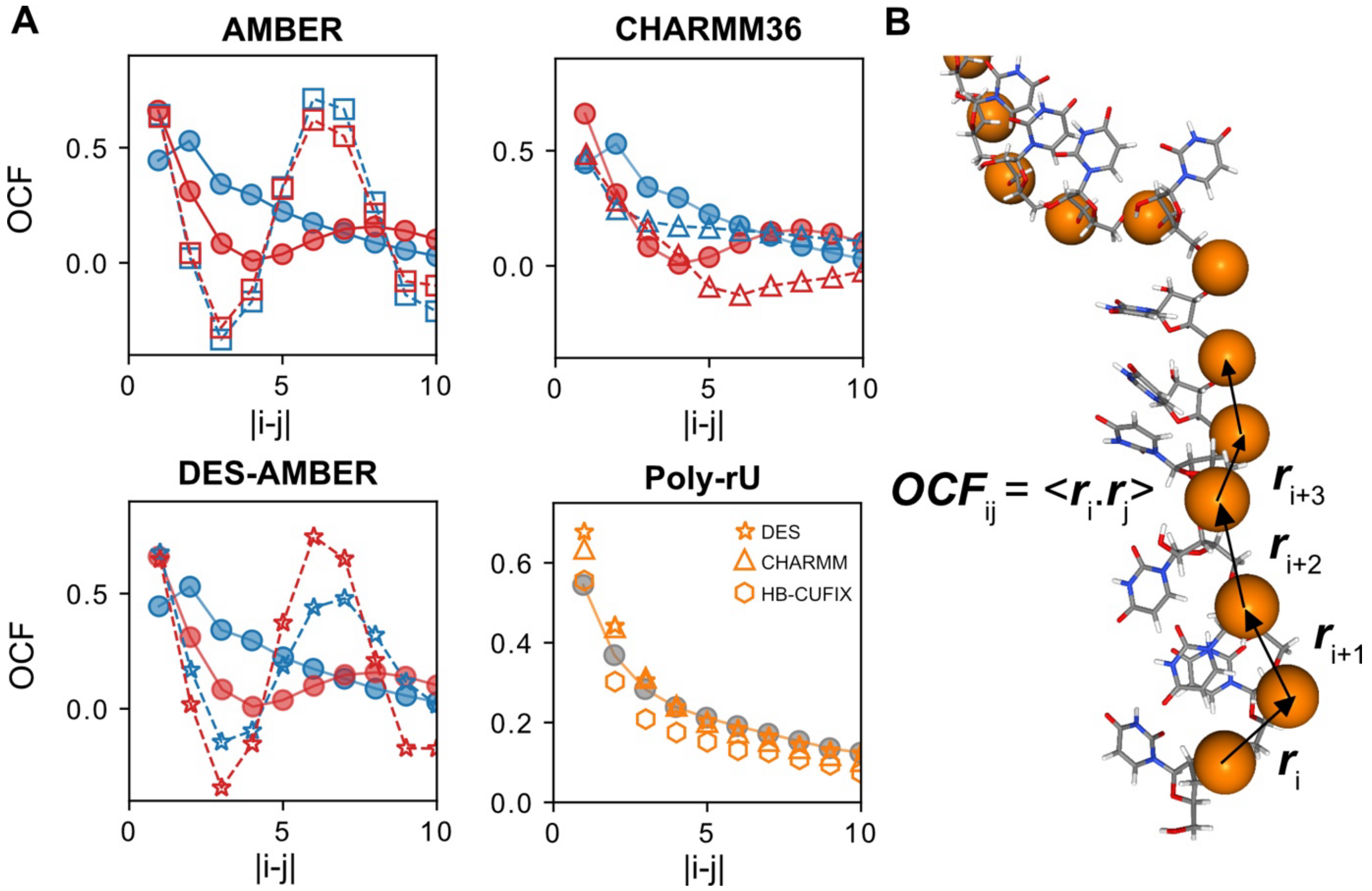


**Fig. S4: Orientational correlation functions (OCF) between ssNA chain phosphates calculated from MD simulation trajectories.** (**A**) OCFs for the $n_{\mathrm{b}}$=30 homopolymers poly-dT – blue, poly-dA – red, poly rU – orange, modelled with the Amberbsc1, CHARMM36, DES-AMBER and HB-CUFIX forcefields (open square, triangle, star and hexagon symbols respectively). Experimentally inferred OCF profiles for $n_{\mathrm{b}}$=30 homopolymers from SAXS data in Refs. [3,4] are presented as solid circular symbols (**B)** Schematic of the calculation of the OCF for an ssNA chain. The OCF is defined as $\langle \hat{r}_i . \hat{r}_j \rangle$, where $(\hat{r}_i . \hat{r}_j)$ are two phosphate site bond vectors in the ssNA chain, separated by $n$ bonds. Phosphate sites are represented as orange spheres.

In modelling poly-rU30, all models considered found good agreement with the experimentally inferred OCF in Ref. 4, with the OCF profiles exhibiting a gradual decay with increased bond separation, whilst still retaining a small positive value of $\langle \hat{r}_i . \hat{r}_j \rangle$ over separations of $|i - j|$ = 10. The DES-AMBER RNA model showed the best agreement with the SAXS data and is a rare example of a model considered in this work that is able to capture both local (OCF) and global (reflected in values of $R_{\mathrm{g}}$ and $R$) polymer chain properties when comparing to the available experimental SAXS and smFRET data.

### S4.2. Conformational landscapes of MD simulated ssNAs

Here we present $R_G$ vs $R$ landscapes for the other ssNA models not shown in Fig. 4 of the main text.

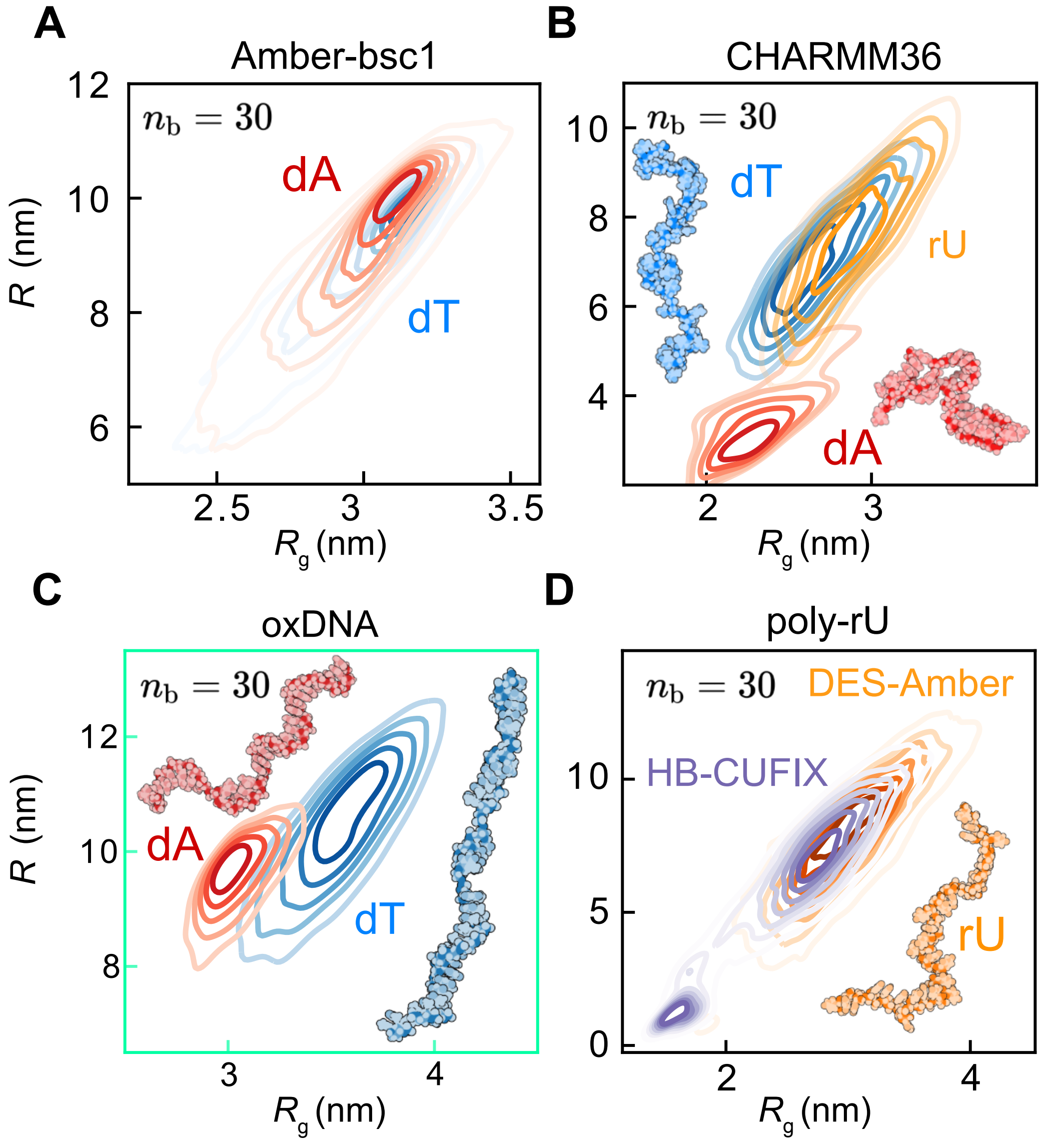


**Fig. S5: Conformational $R_g$ vs $R$ landscapes obtained from MD simulations of ssNAs with different forcefield descriptions** (**A**) Amber-bsc1 poly-dA,-dT for $n_b = 30$ (**B**) CHARMM36 poly-dA,-dT,-rU for $n_b = 30$, (**C**) oxDNA poly-dA,-dT for $n_b = 30$ (**D**) poly-rU comparison with the specialised forcefields DES-Amber and HB-CUFIX for $n_b = 30$. The HB-CUFIX model exhibits a region in conformational space corresponding to very low values of $R_g$ and $R$ due to the formation of hairpin structures. SAXS measurements at 20mM NaCl measure $R_g = 2.96 \pm 0.03$ nm and $2.72 \pm 0.03$ nm for poly-dT30 and poly-dA30 respectively, and an end-to-end distance $R = 7$ nm for both [3].

**S5. Discussion comparing gel electrophoresis and effective charge measurements**

We performed polyacrylamide gel electrophoresis on our $n_{\mathrm{b}} = 30$ and 60 single stranded homopolymeric nucleic acid species (see Fig. 3B and Methods). Echoing the observations in electrometry, the various species migrated differently, despite having the same total structural charge. We observed the following order of increasing electrophoretic mobility: poly-dA > mixed sequence > -dT > -rU (see Fig. 3B). The ordering of mobilities may of course be compared with the magnitude of measured effective charge values. The simplest view of electrophoretic mobility of a charged object in solution suggests $\mu \propto |q_{\mathrm{eff}}|/\xi$, where $\xi$ represents the "Stokes' drag" of the object in the medium. In the Ogston sieving regime, which we may expect to hold for ssDNA oligomers, we expect the mobility to be inversely related to molecular contour length $l_{\mathrm{c}}$ reflecting the inverse relationship between the electrical mobility and Stokes' drag. Since $q_{\mathrm{eff}}$ for poly-dT is larger than that of -rU the electrophoretic mobilities observed for rU and dT may be rationalised based on measured trends in $q_{\mathrm{eff}}$ values alone. However the qualitative trend observed in electrophoretic mobility observed for DNA (poly-dA > mixed sequence > -dT) appears to be the opposite of that indicated by the magnitude of the effective charge. This trend may be explained by base stacking interactions that cause poly-dA to adopt more compact conformations than poly-dT, implying a lower value of $\xi$ for poly-dA [11,12]. Indeed, the observed trend in gel electrophoretic mobilities reflects the order of base-stacking energies reported in the literature, namely AA > mixed > TT > UU [13,14] Nonetheless, for ssDNA it has been demonstrated that molecular affinities for the gel matrix can impact the observed electrical mobility which therefore may not be a solely a function of from physical properties (such as effective charge and stokes drag) that are governed by molecular 3D conformational properties [15]. This disparity between molecular effective charge and electrophoretic mobility has also been noted in a previous study in which pure poly-dT was measured to have the fewest associated excess counterions using AES and hence the largest $|q_{\mathrm{eff}}|$, and yet the smallest mobility when compared to sequences which included a number of other substituted nucleobases [12].

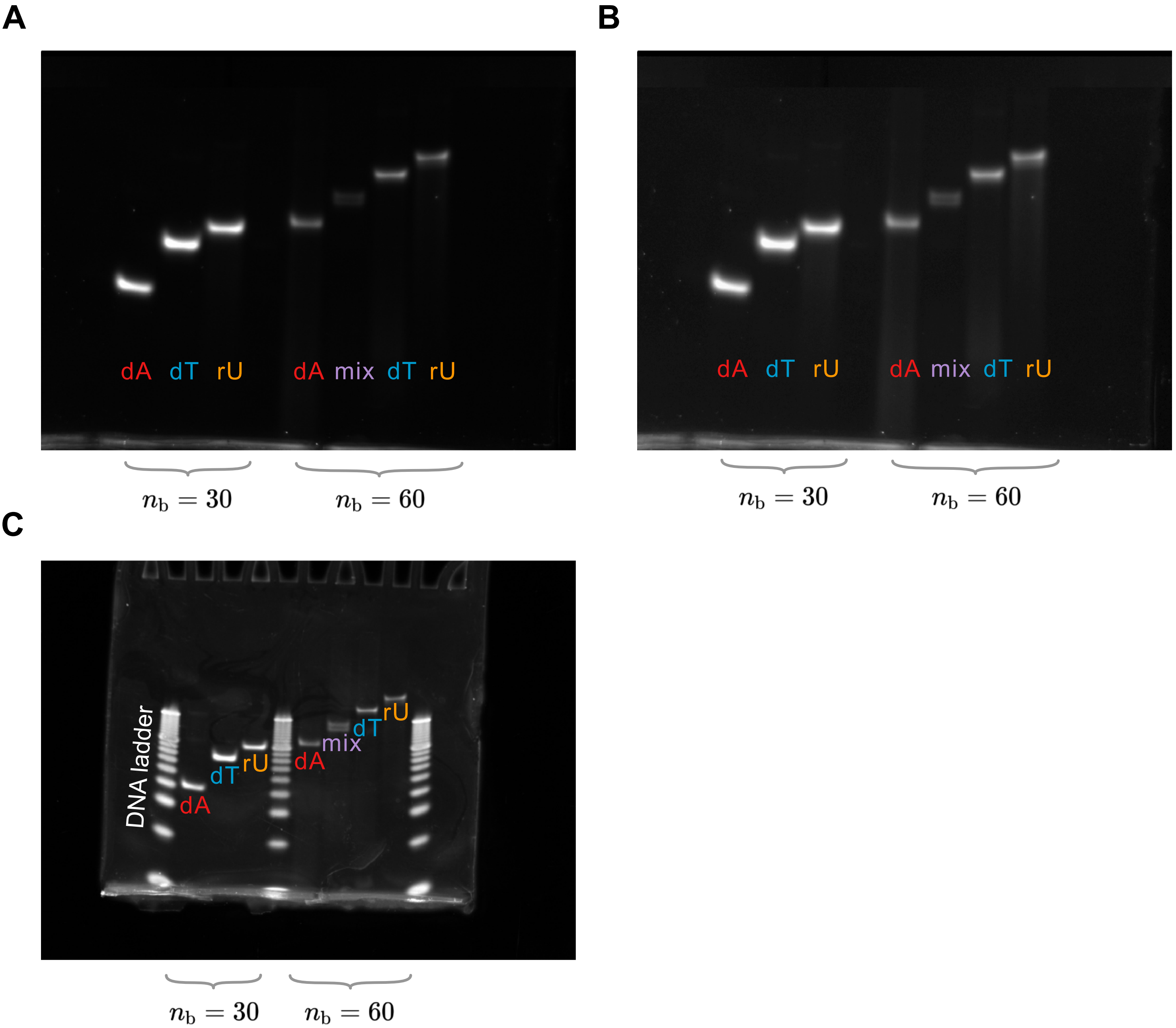


**Fig. S6:** (**A**) Original and (**B**) contrast enhanced full-length gel electrophoresis images of ssNA samples for $n_b$ = 30 and 60, imaged under 532 nm excitation where only the fluorescently labelled DNA, and not the fragments in the molecular weight standard DNA ladder, are visible. Cropped versions of these full-length figures are shown in Fig. 3C. (**C**) Image of the same gel as in (A) and (B) stained with GelRed and visualised to display both the ladder (O'RangeRuler 5 bp DNA Ladder, Thermo Scientific) and the test samples.

**S6. Computational testing of the rod model for ssDNA**

To further test the relationship between the quantities $b$ and $b_c$, we performed a comparison of $q_{\rm calc}$ values determined for molecular models of $n_{\rm b}$= 60 base poly-dT constructed using oxDNA, assuming a width parameter $w$ = 0.2 nm with those determined for rod models of radius $r$ = 0.4 nm and variable $b$. Equating the $q_{\rm calc}$ value in the two models, we obtained a value of the axial charge spacing of $b \approx 0.5$ nm in the rod model that were about 50% smaller than the corresponding average contour length per base of $b_{\rm c} \approx 0.75$ nm inferred both from our molecular-model structures (see Fig. 4D) and also in previous molecular simulation studies [16], thus confirming the qualitatively expected $b < b_{\rm c}$ relationship. This analysis sheds light on the relationship between the values of physically similar parameters obtained from interpreting experimental data using different models of polyelectrolyte conformation.

## S7. On the relationship between the experimental readouts of ET*e* and that of ion counting methods such as atomic emission spectroscopy (AES)

Atomic emission spectroscopy (AES) is a measurement technique that infers an excess ion concentration in the ion atmosphere, $\Gamma$, around the molecule relative to bulk solution. Similar to the effective $q_{\mathrm{eff}}$ as measured by ET*e*, $\Gamma$ also reports on the phenomenon of molecular charge renormalization. In order to make a direct link between these two closely related quantities, we suggested the approximate relationship $\Gamma_{+} \propto a_1 + a_2\eta$, where the coefficients in the equation depend on molecular geometry and salt concentration. In order to deduce this relationship we calculated the counterion excess $\Gamma_{+}$ and charge renormalization factor $\eta$ for rigid rod models of ssDNA with different $r$ and $b$ values (see Fig. S7).

The number of associated ions $i$ of valence $z_i$ associated with a molecule can be computed by solving the Poisson-Boltzmann equation for the molecule immersed in bulk electrolyte, as described in the Methods and Supplementary Information Section S2, by integrating the excess ion number density, as given in Ref. 17:

$$\Gamma_i = c_{\mathrm{i}} N_{\mathrm{A}} \int (\mathrm{e}^{-z_i e\phi/k_{\mathrm{B}}T} - 1)\, dV \qquad \text{(S1)}$$

Indeed, we find that for a highly charged molecule we have a large value of $|\phi|$ resulting in strong charge renormalization which entails both a larger value of excess counterions as well as a lower magnitude of $q_{\mathrm{eff}}$ (smaller $\eta$). Eq. S1 clearly indicates that a large value of $-z_i\phi$ (overall positive for a negatively charged molecule) entails a larger the excess counterion concentration in the molecule's ion atmosphere (see Fig. S7). We note that the quantity $\Gamma_{+}/|q_{\mathrm{str}}|$ (inferred to be $\approx$ 0.71 and 0.74 for our 60 base poly-dT and poly-dA species respectively (see Fig. S7B), can be directly compared with the number of excess counterions per phosphate as reported by AES (reported to be $\approx$ 0.68 and 0.71 for 30 base poly-dT and poly-dA respectively in Ref. 18). Furthermore, the magnitude of the difference in $\Gamma_{+}/|q_{\mathrm{str}}|$ between poly-dA and poly-dT of around 4% can be seen to correspond to about an 11% difference in $\eta$ ($\eta_{\mathrm{A}} = 0.63$ and $\eta_{\mathrm{T}} = 0.70$ as measured by ET*e* for ssDNA species of $n_{\mathrm{b}} = 60$ in this work), highlighting the higher sensitivity of ET*e* compared to AES in the low salt regime (see Fig. S7B), particularly in relation to the relevant measurement precision in each case. It is also worth noting in this context that the indicated relative differences in molecular species

in this example are comparable, or often smaller than, the reported experimental uncertainty in ion counting methods. However these differences are at least one order of magnitude larger than a typical measurement precision in ET*e*.

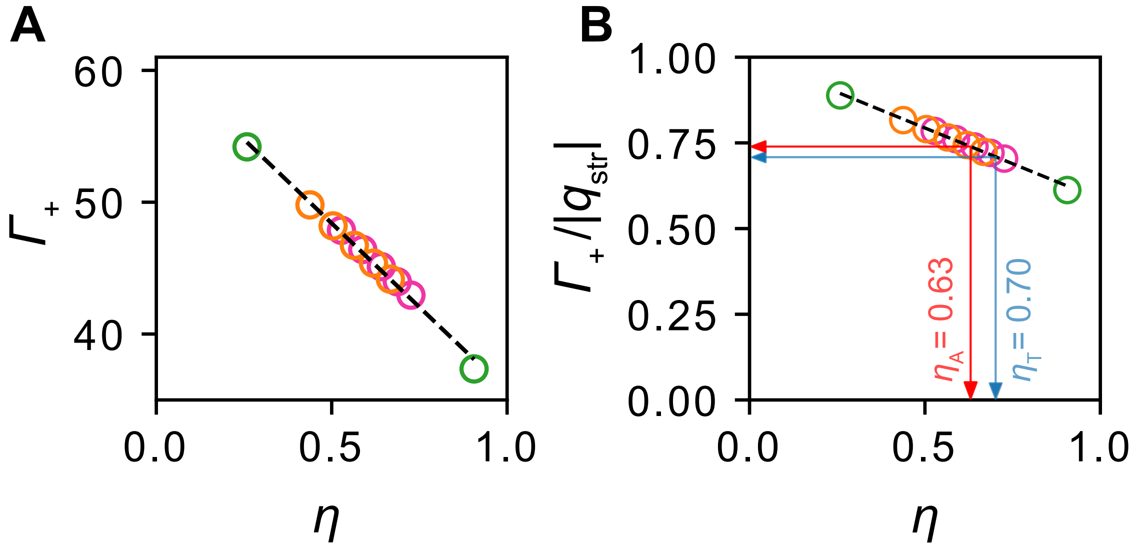


**Fig. S7:** (**A**) Relationship between the counterion excess $\Gamma_+$ and the charge renormalization factor $\eta$ for rigid rod models of ssNAs ($n_b = 60$, $q_{str} = -61\ e$) calculated for our experimental framework as described in Section S2 for a bulk salt concentration $c_0 = 1.2$ mM. Data points are shown for rod models characterised by $r = 0.4$ nm and $b = 0.35 - 0.65$ nm (pink open circles), 'line charge' rod models with $r = 0.05$ nm and $b = 0.35 - 0.65$ nm (orange open circles) and rod models with $r = 0.4$ nm and extreme values of the axial base spacing $b = 0.1$ and $1.4$ nm (green open circles). A linear fit to all data suggests the relationship $\Gamma_+ = -25.441\eta + 61.222$ in the range of rod geometries and at the salt concentration probed (black dashed line). (**B**) Normalised plot of the data presented in (A), where $\Gamma_+$ has been normalised by $|q_{str}|$. 60 base poly-dT and -dA are measured by ET*e* in this work to have average charge renormalization factors $\eta_T = 0.70$ and $\eta_A = 0.63$ respectively. These $\eta$ values correspond $\Gamma_+/|q_{str}|$ values of $\approx 0.71$ and 0.74, shown by red and blue arrows on the ordinate. Thus a $\approx$ 4% difference in the fractional excess counterion values, which is also termed number of $Na^+$ per phosphate in the AES literature, corresponds to an approximately 10% disparity in $\eta$. This is consistent with experimental AES results that reveal correspondingly small differences of $\approx$4% between poly-dT and -dA, and highlights the sensitivity of ET*e* compared to AES in this regime [4,18,19].

**SI References**


1 Graen, T., Hoefling, M. & Grubmüller, H. AMBER-DYES: Characterization of Charge Fluctuations and Force Field Parameterization of Fluorescent Dyes for Molecular Dynamics Simulations. *Journal of Chemical Theory and Computation* **10**, 5505-5512 (2014).

2 Bespalova, M., Behjatian, A., Karedla, N., Walker-Gibbons, R. & Krishnan, M. Opto-Electrostatic Determination of Nucleic Acid Double-Helix Dimensions and the Structure of the Molecule–Solvent Interface. *Macromolecules* **55**, 6200-6210 (2022).

3 Plumridge, A., Meisburger, S. P. & Pollack, L. Visualizing single-stranded nucleic acids in solution. *Nucleic Acids Research* **45**, e66-e66 (2017).

4 Plumridge, A., Andresen, K. & Pollack, L. Visualizing Disordered Single-Stranded RNA: Connecting Sequence, Structure, and Electrostatics. *Journal of the American Chemical Society* **142**, 109-119 (2020).

5 Tucker, M. R., Piana, S., Tan, D., Levine, M. V. & Shaw, D. E. Development of Force Field Parameters for the Simulation of Single- and Double-Stranded DNA Molecules and DNA–Protein Complexes. *The Journal of Physical Chemistry B* **126**, 4442-4457 (2022).

6 Tan, D., Piana, S., Dirks, R. M. & Shaw, D. E. RNA force field with accuracy comparable to state-of-the-art protein force fields. *Proceedings of the National Academy of Sciences* **115**, E1346-E1355 (2018).

7 Liebl, K. & Zacharias, M. The development of nucleic acids force fields: From an unchallenged past to a competitive future. *Biophysical Journal* (2022).

8 He, W., Naleem, N., Kleiman, D. & Kirmizialtin, S. Refining the RNA Force Field with Small-Angle X-ray Scattering of Helix–Junction–Helix RNA. *The Journal of Physical Chemistry Letters* **13**, 3400-3408 (2022).

9 Chen, A. A. & García, A. E. High-resolution reversible folding of hyperstable RNA tetraloops using molecular dynamics simulations. *Proceedings of the National Academy of Sciences* **110**, 16820-16825 (2013).

10 Capobianco, A., Landi, A. & Peluso, A. Duplex DNA Retains the Conformational Features of Single Strands: Perspectives from MD Simulations and Quantum Chemical Computations. *International Journal of Molecular Sciences* **23**, 14452 (2022).

11 Zhang, X. & McGown, L. B. Sequence-based separation of single-stranded DNA at high salt concentrations in capillary zone electrophoresis. *ELECTROPHORESIS* **37**, 2017-2024 (2016).

12 Zhao, J., Cramer, S. M. & McGown, L. B. Mechanism of sequence-based separation of single-stranded DNA in capillary zone electrophoresis. *ELECTROPHORESIS* **41**, 705-713 (2020).

13 Brown, R. F., Andrews, C. T. & Elcock, A. H. Stacking Free Energies of All DNA and RNA Nucleoside Pairs and Dinucleoside-Monophosphates Computed Using Recently Revised AMBER Parameters and Compared with Experiment. *Journal of Chemical Theory and Computation* **11**, 2315-2328 (2015).

14 Šponer, J., Leszczynski, J. & Hobza, P. Electronic properties, hydrogen bonding, stacking, and cation binding of DNA and RNA bases. *Biopolymers* **61**, 3-31 (2001).

15 Stellwagen, N. C. & Stellwagen, E. Effect of the matrix on DNA electrophoretic mobility. *J Chromatogr A* **1216**, 1917-1929 (2009).

16 Andrew, Thomas & Khalid, S. Single-Stranded DNA within Nanopores: Conformational Dynamics and Implications for Sequencing; a Molecular Dynamics Simulation Study. *Biophysical Journal* **103**, 1028-1036 (2012).

17 Gebala, M. & Herschlag, D. Quantitative Studies of an RNA Duplex Electrostatics by Ion Counting. *Biophysical Journal* **117**, 1116-1124 (2019).

18 Plumridge, A., Meisburger, S. P., Andresen, K. & Pollack, L. The impact of base stacking on the conformations and electrostatics of single-stranded DNA. *Nucleic Acids Research* **45**, 3932-3943 (2017).

19 Jacobson, D. R. & Saleh, O. A. Quantifying the ion atmosphere of unfolded, single-stranded nucleic acids using equilibrium dialysis and single-molecule methods. *Nucleic Acids Research* **44**, 3763-3771 (2016).
